# Major chromosome 5H haplotype switch structures the European two-rowed spring barley germplasm of the past 190 years

**DOI:** 10.1007/s00122-023-04418-7

**Published:** 2023-07-21

**Authors:** Ronja Wonneberger, Miriam Schreiber, Allison Haaning, Gary J. Muehlbauer, Robbie Waugh, Nils Stein

**Affiliations:** 1 Leibniz Institute of Plant Genetics and Crop Plant Research (IPK), OT Gatersleben, Corrensstrasse 3, 06466 Seeland, Germany; 2 Division of Plant Sciences, University of Dundee, James Hutton Institute, Invergowrie, Dundee, DD2 5DA Scotland, UK; 3 Department of Agronomy and Plant Genetics, University of Minnesota, St. Paul, MN 55108 USA; 4 Present Address: Cell and Molecular Sciences, James Hutton Institute, Invergowrie, Dundee, DD2 5DA Scotland, UK; 5 School of Agriculture and Wine & Waite Research Institute, University of Adelaide, Waite Campus, Glen Osmond, SA 5064 Australia; 6 Center for Integrated Breeding Research (CiBreed), Georg-August-University, Göttingen, Germany; 7 Present Address: Information and Computational Sciences, James Hutton Institute, Invergowrie, Dundee, DD2 5DA Scotland, UK; 8 Present Address: Department of Pediatrics, Indiana University School of Medicine, Indianapolis, IN USA

## Abstract

**Key message:**

Selection over 70 years has led to almost complete fixation of a haplotype spanning ~ 250 Mbp of chomosome 5H in European two-rowed spring barleys, possibly originating from North Africa.

**Abstract:**

Plant breeding and selection have shaped the genetic composition of modern crops over the past decades and centuries and have led to great improvements in agronomic and quality traits. Knowledge of the genetic composition of breeding germplasm is essential to make informed decisions in breeding programs. In this study, we characterized the structure and composition of 209 barley cultivars representative of the European two-rowed spring barley germplasm of the past 190 years. Utilizing high-density SNP marker data, we identified a distinct centromeric haplotype spanning a ~ 250 Mbp large region on chromosome 5H which likely was first introduced into the European breeding germplasm in the early to mid-twentieth century and has been non-recombining and under strong positive selection over the past 70 years. Almost all cultivars in our panel that were released after 2000 carry this new haplotype, suggesting that this region carries one or several genes conferring highly beneficial traits. Using the global barley collection of the German Federal ex situ gene bank at IPK Gatersleben, we found the new haplotype at high frequencies in six-rowed spring-type landraces from Northern Africa, from which it may have been introduced into modern European barley germplasm via southern European landraces. The presence of a 250 Mbp genomic region characterized by lack of recombination and high levels of fixation in modern barley germplasm has substantial implications for the genetic diversity of the modern barley germplasm and for barley breeding.

**Supplementary Information:**

The online version contains supplementary material available at 10.1007/s00122-023-04418-7.

## Introduction

An understanding of the genetic structure and genetic diversity of crop germplasm is a prerequisite for crop improvement. The availability of diverse germplasm is essential for the maintenance of food security and for improved resilience of crops to abiotic and biotic stresses, especially under climate change (McCouch et al. 2013). Plant breeding, which is based on combining beneficial alleles controlling desirable traits, relies on the availability of wide germplasm pools from which alleles are selected. Over time, breeding has resulted in the constant improvement of crops in terms of yield, disease resistance and improved agronomic and quality traits. A prominent example of this is the Green Revolution and the introduction of semi-dwarfing genotypes in cereals which led to substantial improvements in yield and yield stability (Hedden 2003). However, a constant risk in breeding is the loss of genetic diversity in modern breeding germplasm and hence the loss of beneficial alleles. The consequences are stagnation of the breeding progress and the inability of breeding programs to adapt to different environments or changing market demands (McCouch et al. 2013; Dawson et al. 2015). While repeated intercrossing of elite cultivars or a narrow germplasm pool often leads to a reduction of genetic diversity, it may also generate new beneficial allelic combinations that lead to an improved phenotype (Rasmusson and Phillips 1997). In addition, new genetic diversity may be generated by introducing more exotic alleles into the existing germplasm. In barley (*Hordeum vulgare* L. ssp. *vulgare*), there are contradictory assessments of the overall genetic diversity over time, with some authors reporting a loss of genetic diversity, while most did not observe any significant changes (Donini et al. 2000; Russell et al. 2000; Koebner et al. 2003; Kolodinska Brantestam et al. 2007; Malysheva-Otto et al. 2007; Rajala et al. 2017; Brbaklić et al. 2021). Discrepancies between these studies can likely be attributed to the composition of the population under study, the geographic origin, the time period examined, the relative importance of loss of alleles through selection and the introgression of diverse material. Recent advances in next generation sequencing and high-density marker development have made it possible to not only study genome-wide patterns of genetic diversity but to also identify local changes of genetic diversity over time caused by selection and breeding. For example, the reduced diversity at the *HvCEN* locus on chromosome 2H in European two-rowed spring barleys was the result of selection for late flowering and increased yield associated with this locus (Tondelli et al. 2013). Similarly, the powdery mildew resistance gene *mlo* is now present in most European spring barleys as a result of selection and introgression of resistance alleles from Ethiopian barley (Jørgensen 1992; Friedt et al. 2011).

Barley is the fourth most important cereal crop globally, the second most important crop in Europe and is adapted to a wide range of environmental conditions (Friedt et al. 2011). Domesticated in the Fertile Crescent around 10,000 years ago, barley reached Europe approximately 6–8,000 years ago (von Bothmer et al. 2003; Jones et al. 2011). In Europe, latitudinal adaptation through selection for different flowering times and growth habit allowed the cultivation of barley in diverse day length and climatic environments from Northern Scandinavia to the Mediterranean region. Both spring and winter types are grown in Europe today. The main end uses are animal feed and malt (von Bothmer et al. 2003).

Historically, barley was grown as landraces, i.e. relatively heterogenous populations adapted to local conditions. The first selections from popular spring barley landraces were made in the 1800s in the UK and soon thereafter in other regions of Europe as well (Fischbeck 2003). Among these selections, lines such as ‘Chevalier’ and ‘Goldthorpe’ (selections from British landraces ‘Archer’ and ‘Spratt’, respectively), ‘Hana/Hanna’ (selection from Moravian landraces) or ‘Gull’ (selection from landraces from Gotland, Sweden) became widely successful due to their superior agronomic and malting qualities and were distributed across the continent (Fischbeck 2003; Hagenblad and Leino 2022). At the same time, selections from local landraces were made which were well-adapted to specific environmental conditions and sometimes outcompeted the imported selections but led to a decrease in genetic diversity (Fischbeck 2003). Lines from wider geographic crosses, however, ultimately made up the majority of the successful spring cultivars of that time (e.g. ‘Kenia’ or ‘Isaria’) laying the foundation of modern barley breeding in the mid-twentieth century (Fischbeck 2003; Milotova et al. 2008; Friedt et al. 2011) and leading to the development of widely used varieties such as ‘Golden Promise’ and ‘Triumph’. In the 1950s, crosses became genetically wider by using parents from different breeding programs and more exotic material was introduced such as the landrace *H. laevigatum* which provided resistance to diseases, e.g. powdery mildew (*Blumeria graminis*) and leaf rust (*Puccinia hordei*) (Giese et al. 1993; Hickey et al. 2012). The use of mutation breeding contributed important varieties such as ‘Diamant’ in the 1960s and 1970s which had superior agronomic and malting properties (Friedt et al. 2011). Constant genetic improvement of different agronomic and quality traits has been made over time, although high and stable yield has always been the main focus of barley breeding (Friedt et al. 2011; Laidig et al. 2017; FAOSTAT 2022). In addition, breeding for high protein content in feed barley and low protein content and large grain size, among many other traits, in malting barley has resulted in a range of different varieties suitable for different end uses. The introduction of semi-dwarfing genotypes during the green revolution reduced both plant height and harvest losses due to lodging. Further, yield and quality have been greatly increased by the exploitation of genes conferring different levels of resistance to a range of globally important diseases (Friedt et al. 2011).

Here, we present a high-resolution characterization of barley diversity in a panel of 209 landraces and cultivars representative of 190 years of breeding history in the European two-rowed spring barley germplasm (Schreiber et al. 2023). Based on a high-density SNP dataset comprising 1.5 million SNPs from transcript and whole genome shotgun (WGS) sequencing data derived from the entire diversity panel and a gene expression survey of six tissues sampled during plant development, the genetic and multi-omic characterization allowed us to track major haplotype and transcript changes in the European barley breeding history (Schreiber et al. 2023). To fully exploit this rich resource of high-density multi-omics datasets, we thoroughly studied the genetic and structural aspects of this panel. We characterized population substructure and identified regions exhibiting allelic changes over time, indicative of selection. In particular, we highlight a ~ 250 Mbp pericentromeric and centromeric region on chromosome 5H which has been under exceptionally strong selection during the past 70 years, almost reaching fixation in European two-rowed spring varieties released in the past 20 years. The two main haplotypes in this region differ in the expression of 48 genes and we find evidence of reduced recombination between these haplotypes in the past decades. Utilizing genotypic data from the German Federal ex situ gene bank at IPK Gatersleben we identified North African landraces as a potential geographic origin of this recently preferentially selected haplotype.

## Materials and methods

### Genotypic and expression datasets

The datasets used in this study are described in detail in Schreiber et al. (2023). Briefly, we utilized genotypic and transcript data of 209 barley varieties from the past 190 years which were selected for their representativeness of the European two-rowed spring barley germplasm (Schreiber et al. 2023, Online Resource 1). The genotypic dataset comprised 1,509,446 biallelic SNP markers derived from 150 bp paired-end RNAseq data from six tissues (seedling crown, seedling root, peduncle, developing inflorescence, spikelet at green anther stage and developing grain five days post anthesis), WGS skim sequencing at threefold haploid genome coverage and the 50 k Illumina Infinium iSelect array (Bayer et al. 2017). The SNPs were filtered for < 2% heterozygosity and minor allele frequency (MAF) > 0.025. Missing data were imputed using a haplotype-based approach implemented in Tassel 5.0 (Bradbury et al. 2007) and SNPs were pruned using an LD cutoff of 0.99 in Plink v1.9 (Purcell et al. 2007). The gene expression datasets were constructed from the same RNAseq data after pseudo-alignment against the barley reference transcriptome BaRTv2.0 using Salmon v.1.3.0 (Patro et al. 2017; Coulter et al. 2022). Expression was calculated as log-transformed counts per million (cpm). Unless otherwise stated, all physical positions of genetic features refer to the reference genome of ‘Barke’, a European two-rowed spring barley variety (Jayakodi et al. 2020).

### Population genetics analyses

To visualize genetic patterns over time, a Discriminant Analysis of Principal Components (DAPC) was performed on the panel clustered by release period (1830–1959, 1960–1969, 1970–1979, 1980–1989, 1990–1999, 2000–2009, 2010–2014) using the function ‘dapc’ from the R package ‘adegenet’ v.2.1.4 and 600,000 randomly selected SNPs (Jombart 2008). The 'adegenet' function ‘xvalDapc’ was used to perform a cross-validation with 100 repetitions to identify the optimal number of PCs to retain for the analysis.

To visualize genetic variation and population structure, a Principal Coordinate Analysis (PCoA) was performed using all 1,509,446 SNPs in the dataset. A neighbor-joining tree was created based on a distance matrix using the ‘nj’ function in the R package ‘ape’ v.5.6–1 (Paradis and Schliep 2019). To select the number of principal components required to explain ~ 90% of the variance in the population and to identify the most likely number of subpopulations and to assign accessions to these subpopulations, k-means clustering using the function ‘find.cluster’ from the R package ‘adegenet’ v.2.1.4 was used with 10,000 iterations. Due to limited computational resources, the clustering of the European two-rowed spring barley panel was done on a subset of 1,000,000 randomly selected SNPs.

The polymorphism information content (PIC) was calculated in rolling windows of 10,000 SNPs using the following equation (Botstein et al. 1980):

$$PIC = 1 - \sum\limits_{{i = 1}}^{n} {p_{i}^{2} } - \sum\limits_{{i = 1}}^{{n - 1}} {\sum\limits_{{j = i + 1}}^{n} {2p_{i}^{2} p_{j}^{2} } }$$

with p_i_ and p_j_ being the frequency of the *i*th and *j*th allele, respectively and* n* the number of markers. Frequencies of reference alleles (of cultivar ‘Barke’) were calculated in rolling windows of 10,000 SNPs.

FST (Weir and Cockerham 1984) between subpopulations was calculated in 100 kbp windows with a step size of 10 kbp using the software vcftools 0.1.16 (Danecek et al. 2011). Pairwise FST between cultivar groups from different release periods was calculated using the ‘pairwise.WCfst’ function from the ‘hierfstat’ package v.0.5–10 (Goudet 2005). Variance within and between subgroups defined by release period was calculated using analysis of molecular variance (AMOVA) as implemented in the ‘poppr.amova’ function in the R package ‘poppr’ (Kamvar et al. 2014) and a subset of 400,000 randomly selected SNPs.

### Haplotype characterization

Two major haplotypes were apparent in the centromeric region of chromosome 5H through FST analysis. Utilizing the gene expression data of six tissues, we studied patterns of gene expression in the genes located in this region and tested whether gene expression is haplotype-dependent. A PCoA of the log-transformed cpm values was performed on the cultivars to identify clusters of individuals with similar patterns of gene expression and to look for overlaps between gene expression clusters and haplotype clusters. Next, a PCoA of the log-transformed cpm values was performed on the genes and combined with a k-means cluster analysis to cluster genes according to their expression profiles across cultivars. Expression of genes with haplotype-dependent expression was visualized across all cultivars and tissues in a heatmap using the ‘pheatmap’ package in R. Dotplots of genome alignments were made using a custom R script utilizing lastz 1.04.03 (Harris 2007). GO term enrichment analysis was performed with the R package ‘topGO’.

### Haplotype analysis in the collection of the German Federal ex situ gene bank at IPK Gatersleben

The origin and geographic distribution of the haplotype present in the cluster of more recent cultivars was analysed in a collection of approximately 20,000 domesticated barley accessions stored in the German Federal ex situ gene bank at IPK Gatersleben for which high-resolution SNP datasets generated by genotyping-by-sequencing (GBS) and morphological and agronomic passport data are available (Mascher, pers. comm.; Milner et al. 2019). Since the SNP dataset was mapped to the third version of the reference genome of cultivar ‘Morex’ (Mascher et al. 2021), we blasted 5000 bp sequences flanking the region of interest on chromosome 5H of cultivar ‘Barke’ (as identified by high FST values) against the ‘Morex’ V3 genome (67–320 Mbp on chromosome 5H) and extracted the SNPs in this region. The SNP dataset was filtered for < 20% missing data and a minor allele count of 1, resulting in 6536 SNP markers in the region. Using the *k*-means clustering approach described above we identified the number of clusters that sufficiently described the structure in the dataset and performed a PCoA on the domesticated barley accessions. Based on the cluster membership of accessions with known haplotypes (from the panel of 209 European cultivars), we identified gene bank accessions belonging to the cluster corresponding to the newer haplotype. Maps showing the distribution of this haplotype by country and different passport data categories (e.g. cultivation status, row type etc.) were made using the R package ‘maps’. Countries for which fewer than 15 accessions per respective category were present were excluded.

## Results

### Genetic changes in the population over time

To characterize genetic changes in the population over time, we divided the population into different groups according to release year, either in periods of 10 years or 20 years from 1960 onwards with landraces and cultivars released before 1959 forming a separate group.

**Fig. 1:**
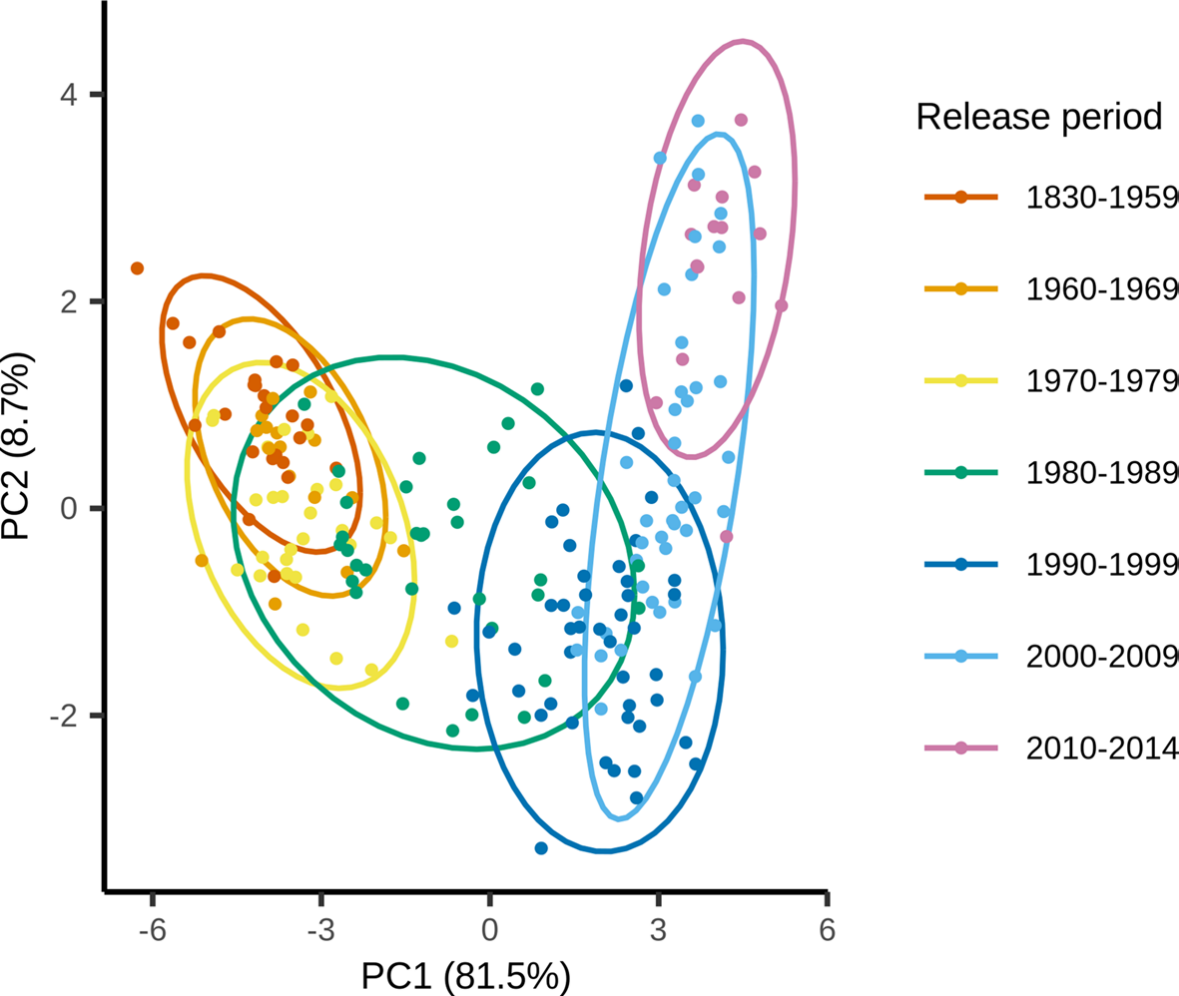
Discriminant Analysis of Principal Components (DAPC) of the European two-rowed spring barley panel with pre-defined groups according to release period using 600,000 randomly selected SNPs. Principal component (PC) 1 represents a development of the population over time. Increased variation between and within more recent clusters suggests an increase in genetic diversity after the 1980s

Changes based on release year along the first axis were apparent in the DAPC analysis (Fig. 1). Cultivars with a greater difference in release year tended to be located further apart than cultivars released at a similar time. The clusters including cultivars released before 1980 showed considerable overlap, while the newer clusters showed more variation within and between clusters suggesting an increase in genetic diversity. These observations are reflected in pairwise FST values between the groups based on release period, which tended to increase with temporal distance between time periods (Online Resource 2). Most of the genetic variation in the panel was found between samples within a release period (86%), whereas smaller fractions were attributed to variation between release periods (14%) (Online Resource 3).

**Fig. 2:**
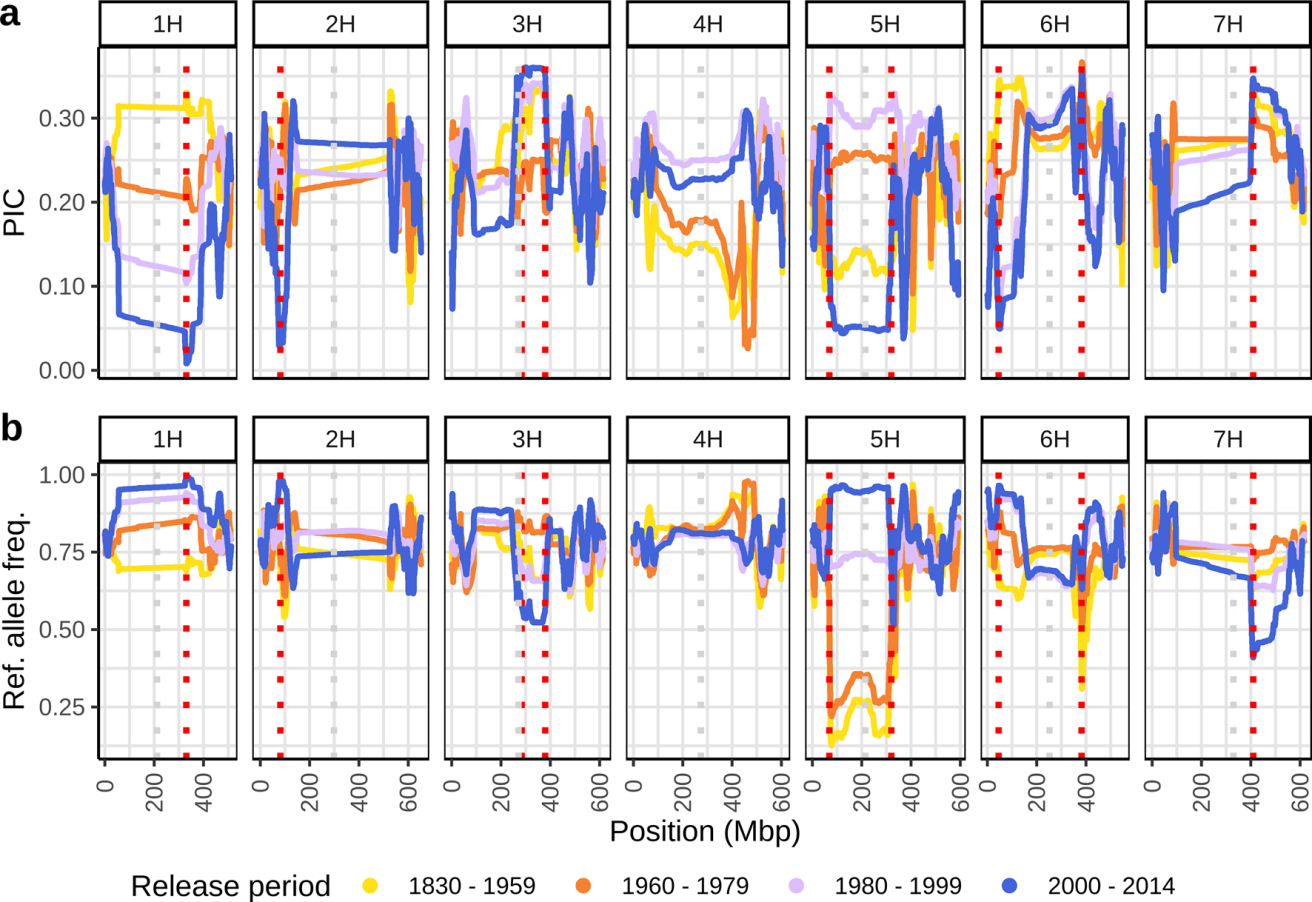
Polymorphic information content (PIC) (**a**) and frequencies of the reference alleles (from cultivar ‘Barke’) (**b**) in rolling windows of 10,000 SNPs in cultivar groups based on release period. Regions of interest are highlighted by vertical red dotted lines and described in the text. The pericentromeric and centromeric region on chromosome 5H (69–320 Mbp) is characterized by low PIC values in early and recent cultivar groups and a strong increase in reference allele frequency in cultivar groups released after the 1980s, providing evidence for a change in haplotype over time. Centromeric positions were estimated by mapping centromeric Morex V3 genes to Barke and are highlighted by vertical grey dotted lines (color figure online)

PIC values differed greatly between release periods in the pericentromeric and centromeric region of chromosome 5H, which showed very low PIC values in groups of cultivars released before 1960 and after 2000 and higher PIC values in the cultivar groups released between 1960 and 2000 (Fig. 2a). Reference allele frequencies increased continuously over time in this region: the groups of cultivars released before 1979 were characterized by reference allele frequencies between 0 and 0.36, whereas the group of cultivars released between 1980 and 1999 had reference allele frequencies around 0.75 and the cultivar group released after 2000 had reference allele frequencies between 0.89 and 0.96 (Fig. 2b). Thus, older cultivars have a different haplotype that has decreased in frequency over time whereas modern cultivars released after 2000 almost exclusively carry the same haplotype as the reference cultivar ‘Barke’.

Another region in which PIC values varied greatly was identified at 330.42 Mbp on chromosome 1H. PIC values decreased over time from 0.33 in the group of cultivars released before 1959 to almost reaching zero in the group of cultivars released after 2000 (Fig. 2a). This development was due to an increase of the ‘Barke’ allele over time, which reached almost complete fixation among the newest cultivars in our panel (Fig. 2b). The entire pericentromeric and centromeric region of chromosome 1H, generally a region of low diversity in spring barleys (Mascher et al. 2017; Schreiber et al. 2023), was characterized by an increase of the ‘Barke’ allele over the last decades.

Similarly, the newest cultivar group showed low PIC values at 81.4 Mbp on chromosome 2H and 45.6 Mbp on chromosome 6H (Fig. 2a). These regions have potentially been subjected to a decrease in genetic diversity in breeding programs in recent years as frequencies of the ‘Barke’ alleles of > 0.95 were observed in the newest cultivars whereas these alleles were present at much lower frequencies in the older cultivar groups (Fig. 2b). Conversely, regions with high PIC values on chromosome 3H, 6H and 7H in the more recent groups may have experienced recent introgressions from other germplasm sources, which is further supported by a drop in reference allele frequency, suggesting that new alleles not present in ‘Barke’ have been recently introduced to the germplasm (Fig. 2).

### Clustering analysis and population structure

**Fig. 3:**
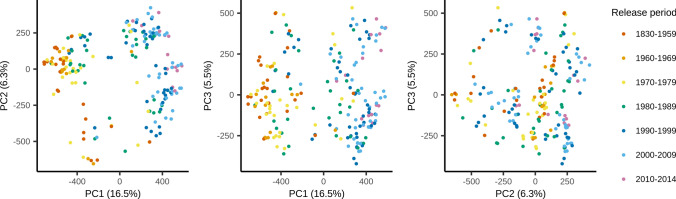
Principal coordinate analysis (PCoA) of the European two-rowed spring barley panel using the genotypes of 1,509,447 SNP markers. Each dot represents a cultivar, color-coded according to the period in which they were registered as commercial cultivars

In addition to the a priori clustering of cultivars by release year described above, overall population structure was analysed using several different approaches. The PCoA (Fig. 3) and the neighbor-joining tree (Online Resource 4) suggest a moderate degree of clustering into two groups which to some extent is attributable to the release year of the cultivars. The first principal coordinate (PCo1) placed the cultivars on a gradient corresponding to the release year, explaining 16.5% of the total variation and the neighbour-joining tree clustered most of the older cultivars released before 1990 into a separate group which included only few cultivars released after 1990. A clustering according to geographic origin was not apparent (Online Resource 5). In addition, we used k-means clustering to identify the most likely number of subpopulations. Approximately 110 principal components (PCs) were required to capture ~ 90% of the variation in the dataset, underlining the lack of major structure in the population (Online Resource 6). With 110 PCs retained the lowest Bayesian information criterion (BIC) value, an indicator of the best fit was reached at *k* = 9 clusters. Given the lack of strong population structure observed in the PCoA, such a high number of subpopulations seemed unlikely. Hence, and since we were interested in the main drivers of population differentiation, we followed the ‘elbow method’ instead and concluded that two or three clusters would provide a reasonable description of the data (Online Resource 4, Online Resource 7). Assuming two subpopulations, the panel was divided into a slightly older (cluster 1, most cultivars released before 1990) and a slightly younger population (cluster 2, most cultivars released after 1980) (Table 1, Online Resource 1, Online Resource 7), coinciding with the clusters apparent in the PCoA (Fig. 3). At *k* = 3, the cluster of more recent cultivars was further divided into two clusters consisting of cultivars of similar age and geographic origin (clusters 2 and 3) (Table 1, Online Resource 1, Online Resource 7). Two cultivars that belonged to the older cluster at *k* = 2 were assigned to one of the younger clusters (cluster 3) at *k* = 3.

**Table 1:** Summary table of the geographic and temporal composition of the k-means clusters at *k* = 2 and *k* = 3. Numbers indicate the number of cultivars per respective category

Country	*k* = 2		*k* = 3			Total
	Cluster 1	Cluster 2	Cluster 1	Cluster 2	Cluster 3	
Czech Republic	3	5	3	1	4	8
Denmark	14	6	14	3	3	20
Finland	4	0	4	0	0	4
France	4	8	4	6	2	12
Germany	11	13	11	5	8	24
Italy	2	0	2	0	0	2
Latvia	2	0	2	0	0	2
Netherlands	8	6	7	3	4	14
Norway	1	0	1	0	0	1
Slovakia	1	0	1	0	0	1
Sweden	17	2	17	0	2	19
UK	18	71	17	35	37	89
Unknown	0	5	0	3	2	5
Release period						
1830–1959	23	1	23	1	0	24
1960–1969	15	1	15	0	1	16
1970–1979	22	6	22	3	3	28
1980–1989	15	18	15	7	11	33
1990–1999	9	35	7	18	19	44
2000–2009	1	40	1	20	20	41
2010–2014	0	15	0	7	8	15
Total	85	116	83	56	62	201

**Fig. 4:**
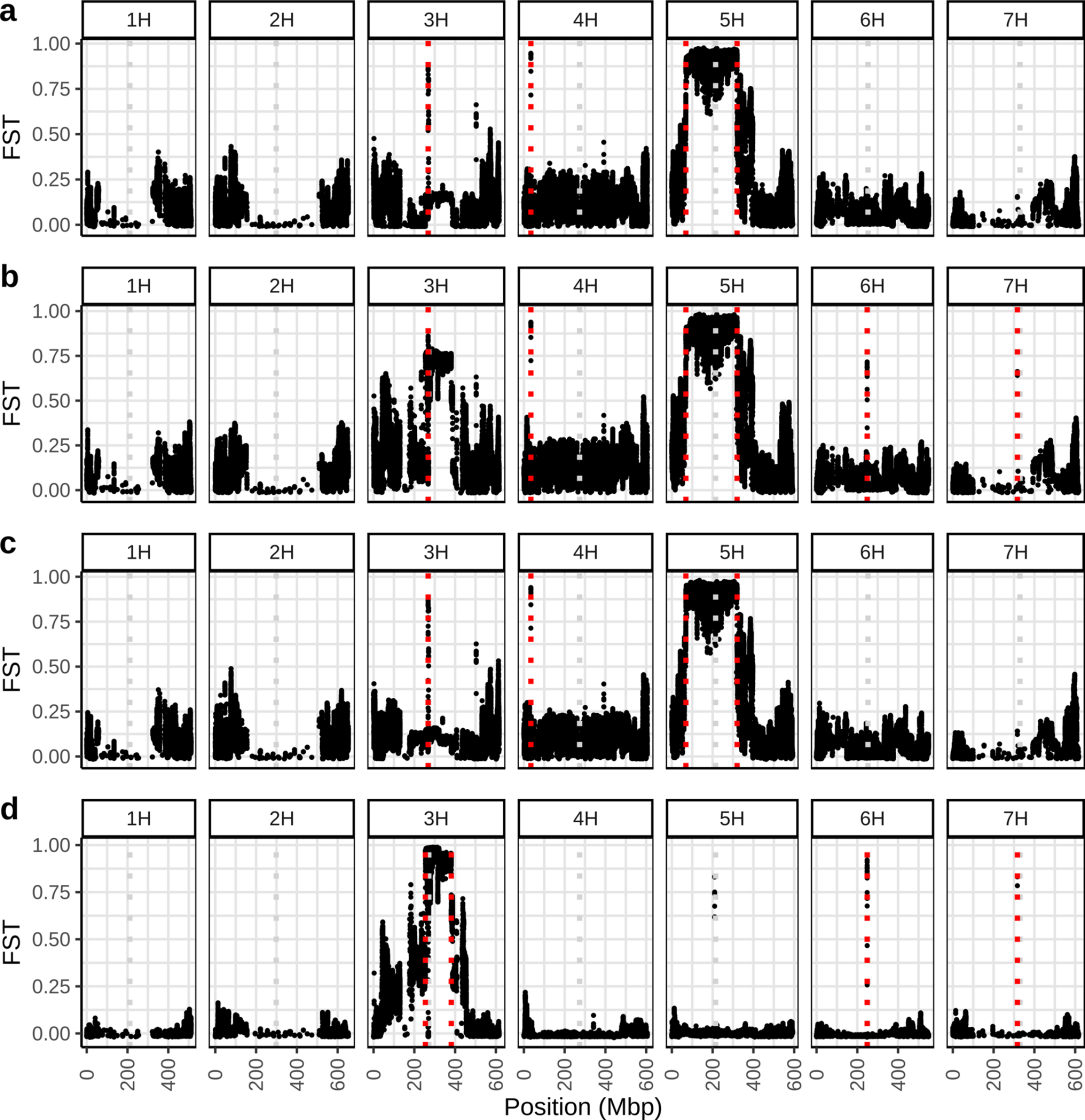
FST between clusters in 100 kbp windows with a step size of 10 kbp along the genome between **a** clusters 1 and 2 at *k* = 2, **b** clusters 1 and 2 at *k* = 3, **c** clusters 1 and 3 at *k* = 3, and **d** clusters 2 and 3 at *k* = 3. Clusters were determined by *k*-means clustering under the assumption of two or three clusters, respectively. Regions with high FST values between different clusters are highlighted by vertical red dotted lines and described in the text. The pericentromeric and centromeric region on chromosome 5H (68–320 Mbp) is characterized by high FST values between the two clusters at *k* = 2 as well as between clusters 1 and 2 and 1 and 3 at *k* = 3 respectively. FST values of 0.7–1 in this region suggest almost complete fixation of different haplotypes in the different clusters. Centromeric positions were estimated by mapping centromeric Morex V3 genes to Barke and are highlighted by vertical grey dotted lines (color figure online)

**Fig. 5:**
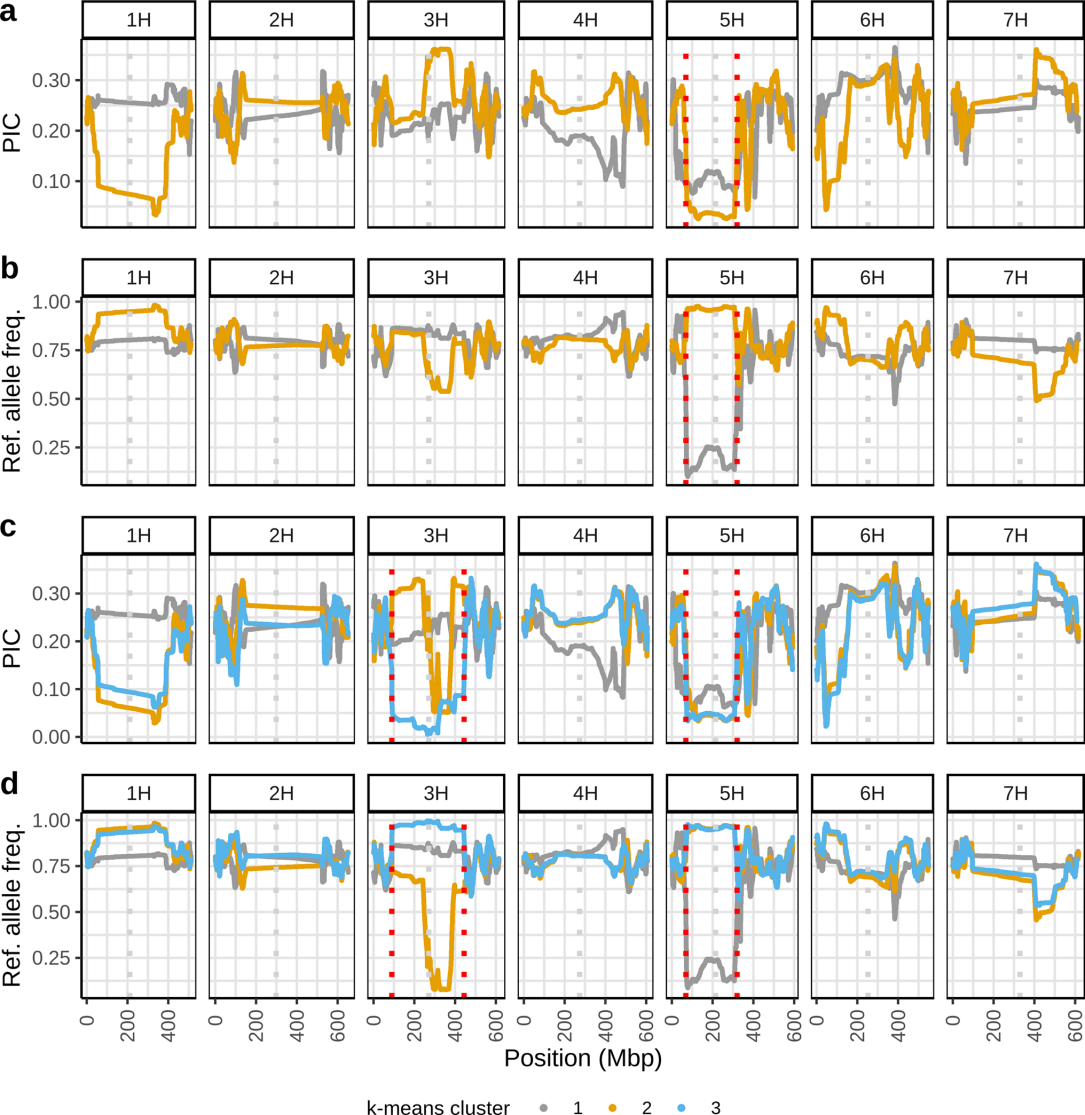
Polymorphic information content (PIC) (**a**,** c**) and frequencies of the reference alleles (from cultivar ‘Barke’) (**b**,** c**) in rolling windows of 10,000 SNPs in the different k-means clusters at* k* = 2 (**a**,** b**) and* k* = 3 (**c**,** d**). Regions of interest are highlighted by vertical red dotted lines and described in the text. The pericentromeric and centromeric region on chromosome 5H (69–320 Mbp) is characterized by low PIC values in all clusters. Low reference allele frequencies in older cultivars (cluster 1 at* k* = 2 and* k* = 3) and high reference allele frequencies in newer cultivars (cluster 2 at* k* = 2 and clusters 2 and 3 at*k* = 3) illustrate the haplotype change over time in this region. Centromeric positions were estimated by mapping centromeric Morex V3 genes to Barke and are highlighted by vertical grey dotted lines (colour figure online)

FST analysis detected genomic regions characterized by different allele frequencies in these clusters (Fig. 4). FST values above 0.7 between 68.78 and 320.04 Mbp on chromosome 5H indicate very different allele frequencies and therefore support the presence of different haplotypes in this region in the two clusters at *k* = 2 (Fig. 4a). This is confirmed by low PIC values in this region and low reference allele frequencies in the old cluster (cluster 1) and high reference allele frequencies in the new cluster (cluster 2) (Fig. 5a and b). In addition, FST values above 0.8 were found at 267 Mbp on chromosome 3H and at 34 Mbp on chromosome 4H (Fig. 4a). At *k* = 3, the regions on chromosomes 5H, 3H and 4H were associated with FST values above 0.7 between clusters 1 and 2 and 1 and 3, respectively (Fig. 4b and c). In addition, loci with FST values above 0.6 at 248 Mbp on chromosome 6H and at 316 Mbp on chromosome 7H were apparent between clusters 1 and 2 (Fig. 4b). The differentiation between the two newer clusters 2 and 3 was due to an almost complete fixation for opposite alleles between 254.2 and 381.1 on chromosome 3H (FST > 0.8 in most 10 kbp windows in the region) in addition to small regions on chromosomes 5H, 6H and 7H (Fig. 4d). While cluster 3 is almost fixed for the ‘Barke’ haplotype in the region on chromosome 3H, cluster 2 is almost fixed for a different haplotype (Fig. 5c and d). Interestingly, the region of fixation in cluster 3 is larger than in cluster 2. The genomic regions at 267 Mbp on chromosome 3H, at 248 Mbp on chromosome 6H and at 316 Mbp on chromosome 7H coincided with the approximate centromere positions and may be caused by mapping errors to these highly repetitive regions.

### Analysis of haplotypes and gene expression on chromosome 5H

**Fig. 6:**
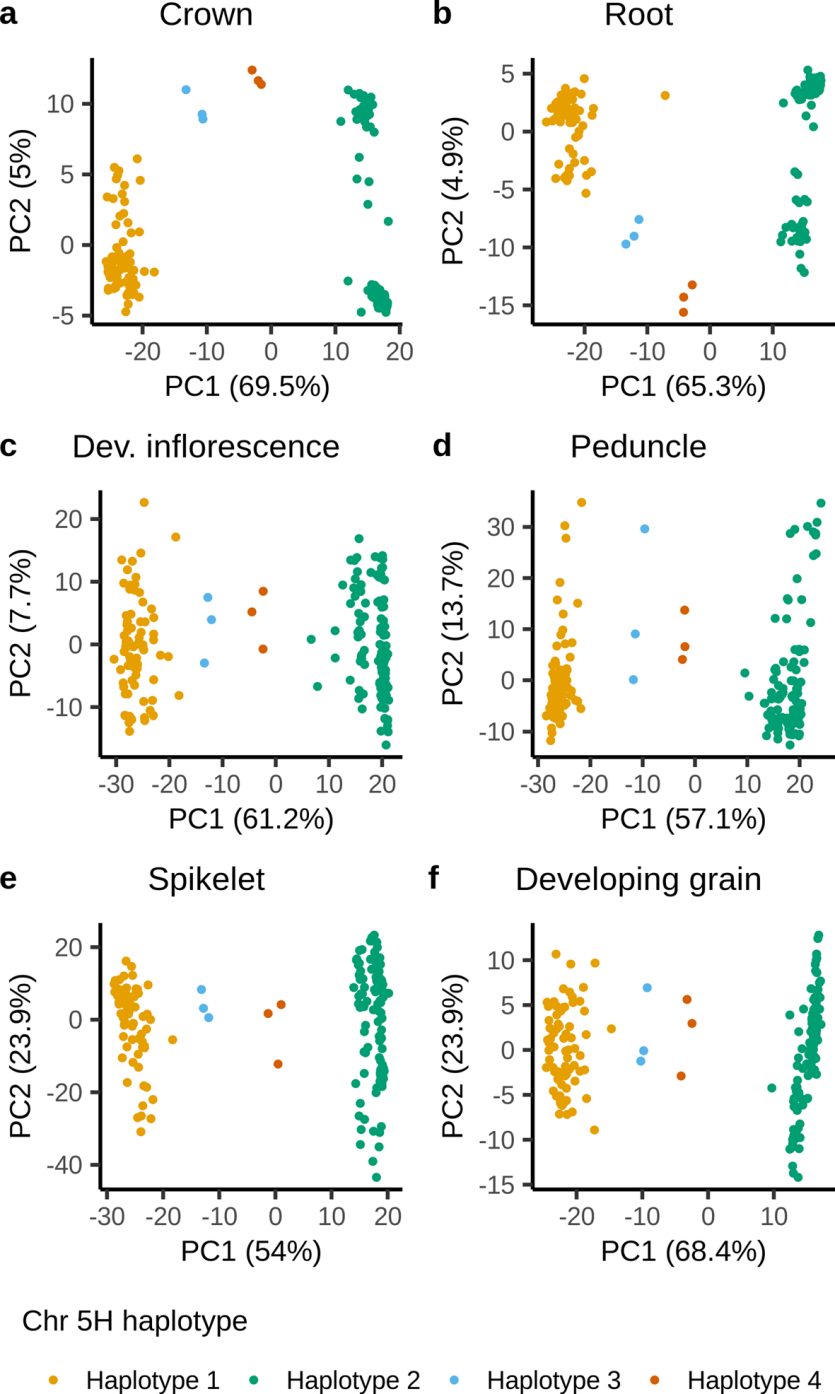
Principal coordinate analysis (PCoA) of the expression of the 827 genes in the haplotype region on chromosome 5H in six tissues. Cultivars are color-coded according to the haplotype they carry

**Fig. 7:**
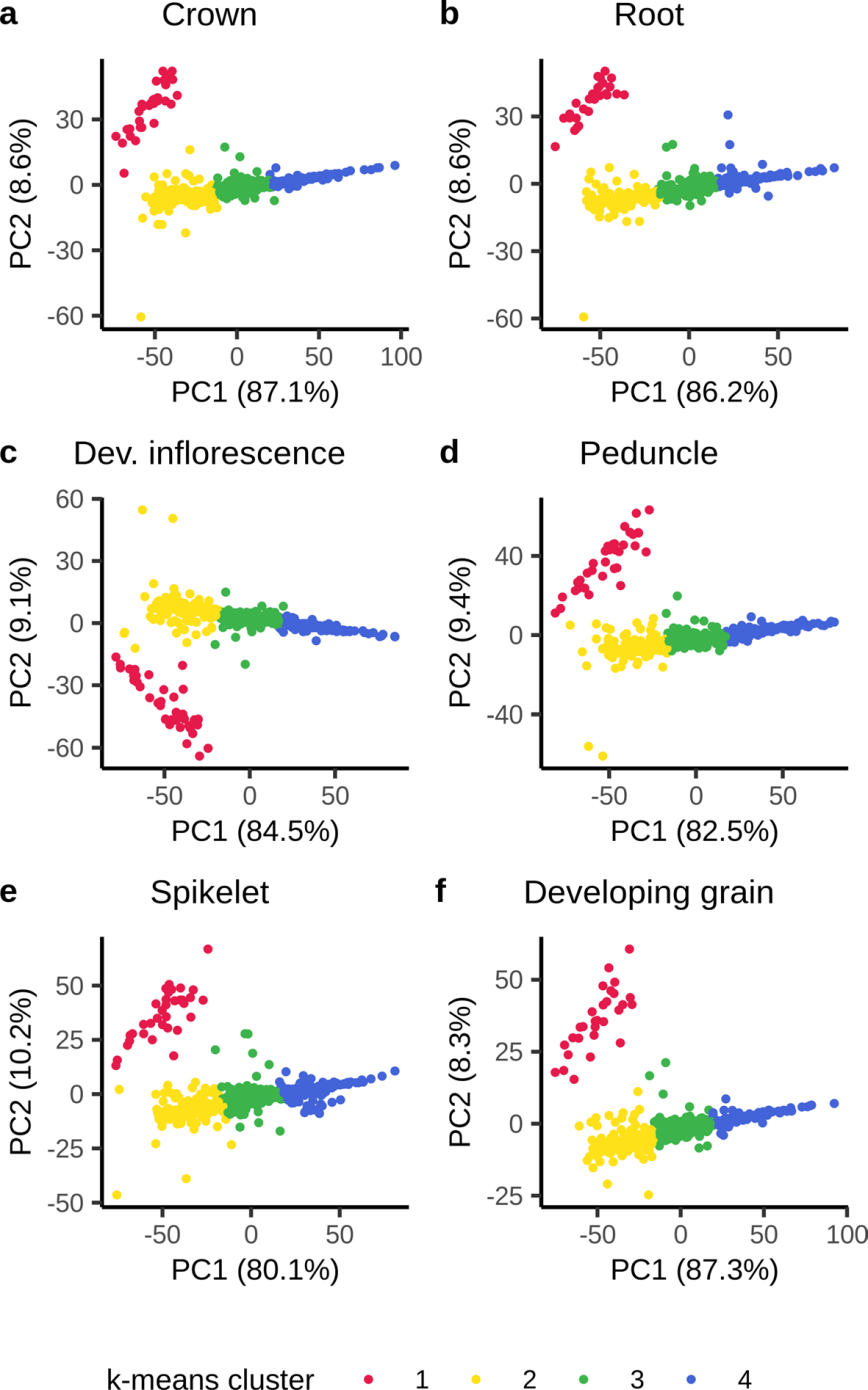
Principal coordinate analysis (PCoA) of the expression of the 827 genes in the haplotype region on chromosome 5H in six tissues. Each dot represents a gene, color-coded by the k-means cluster they were assigned to under the assumption of the presence of four distinct gene clusters (*k* = 4). A group of in total 46 genes (red) is apparent along PC2 which does not cluster with the rest of the genes in this region. These are the genes which are up-regulated in haplotype 2. In addition, one or two genes cluster separately on the opposite end of PC2 from the red cluster. These are the genes which are down-regulated in haplotype 2 (color figure online)

In the chromosome 5H region characterized by high FST values between the older cluster 1 and the more recent cluster 2 (at *k* = 2), including the flanking 10 kbp, 1080 BaRTv2.0 gene models are present, of which 827 had an expression of > 1 cpm in > 30% of the cultivars in at least one of the six tissues. A PCoA of the log-transformed cpm expression values of these genes separated the cultivars into the two k-means clusters along the first PC in all six tissues (Fig. 6, orange and green groups); however, six cultivars (‘Georgie’, ‘Vada’, ‘Reggae’, ‘Abacus’, ‘Apex’ and ‘Livet’) formed two separate clusters between the two main haplotype groups. Across all six tissues we identified 46 genes which formed a separate cluster (red cluster, Fig. 7, Online Resource 8) whose expression was strongly haplotype-dependent (Online Resources 9–14). In the haplotype predominant in older cultivars (henceforth referred to as haplotype 1) these genes were characterized by no or low expression, whereas in cultivars carrying the new haplotype (haplotype 2) the genes were moderately to highly expressed (Online Resource 9–14). In addition, we identified two genes highly expressed in cultivars carrying haplotype 1 and lowly expressed in cultivars carrying haplotype 2. These patterns were consistent across all six tissues. In the six cultivars forming two distinct clusters, expression of these genes was more variable and did not follow the described pattern. Here, two patterns in gene expression were apparent with cultivars ‘Georgie’, ‘Vada’ and ‘Abacus’ forming one group and ‘Reggae’, ‘Apex’ and ‘Livet’ forming another group. ‘Georgie’ and ‘Abacus’ are derived from crosses with ‘Vada’ which is a cross between ‘Gull’ and *H. laevigatum*. A PCoA of the 119,914 SNPs in the region placed the six cultivars into the same two distinct clusters between haplotype 1 and 2, confirming that these cultivars indeed carry distinct haplotypes (Online Resource 15). We define the *H. laevigatum*-derived haplotype as haplotype 3 and the haplotype found in ‘Reggae’, ‘Apex’ and ‘Livet’ as haplotype 4.

The pronounced changes of FST values at the beginning and at the end of the 5H region and graphical genotypes of 10,000 randomly selected markers (Fig. 4, Online Resource 16, Online Resource 17) illustrate the distinctness and the lack of recombination between haplotype 1 and 2. Except for cultivars ‘Novello’, ‘Heron’, ‘Fairytale’, ‘Chieftain’, ‘Berenice’, ‘Nomad’, ‘Kym’ and ‘Drost’ the start of the haplotype block is very conserved (Online Resource 17a and c), and the end of the haplotype block is conserved in all cultivars (Online Resource 17b and 17d). No signs of recombination are apparent. Of the recently published 20 reference genomes representing the first version of a barley pan-genome (Jayakodi et al. 2020) we identified haplotype 1 in the accessions and cultivars ‘Golden Promise’, ‘Morex’, ‘HOR 13821’, ‘HOR 3081’ and ‘HOR 3365’ and haplotype 2 in ‘Barke’, ‘Igri’, ‘HOR 13942’ and ‘RGT Planet’. Genome alignments show an insertion at the beginning of the haplotype block in ‘Barke’ and ‘RGT Planet’ (ca. 61–68 Mbp in ‘Barke’) which is not present in accessions and cultivars carrying haplotype 1, as well as two inversions at 79–84 Mbp (Online Resources 18a and b, Online Resource 19). In addition, the end of the haplotype block is characterized by an insertion in haplotype 2 (ca. 312–316 Mbp in ‘Barke’) followed by a potential translocation of a haplotype 2 segment further upstream into haplotype 1 (Online Resources 18a and c).

A Blast search showed that the genomic region of BaRT2v18chr5HG229090 at 81.11 Mbp was not found in any of the accessions carrying haplotype 1 in the pan-genome but is present in haplotype 2 accessions. In addition, BaRT2v18chr5HG238070, BaRT2v18chr5HG238380, BaRT2v18chr5HG238390, BaRT2v18chr5HG238400, BaRT2v18chr5HG238410, BaRT2v18chr5HG238630, BaRT2v18chr5HG238640 at 307.73–317.47 Mbp at the end of the haplotype region are absent in haplotype 1 genomes. BaRT2v18chr5HG238320 (311.83 Mbp), BaRT2v18chr5HG238440 and BaRT2v18chr5HG238450 (315.86–315.88 Mbp) showed Blast hits further downstream in haplotype 1 accessions, suggesting that they are present on the translocated segment.

A GO enrichment analysis of the 1080 genes located between 68.78 and 320.04 Mbp in chromosome 5H identified six significantly enriched terms suggesting an involvement of these genes in general processes such as calmodulin and abscisic acid binding, phosphatase activity and salicylic acid biosynthesis processes (Online Resource 20).

### Occurrence of the new haplotype 2 in a global barley germplasm collection

**Fig. 8:**
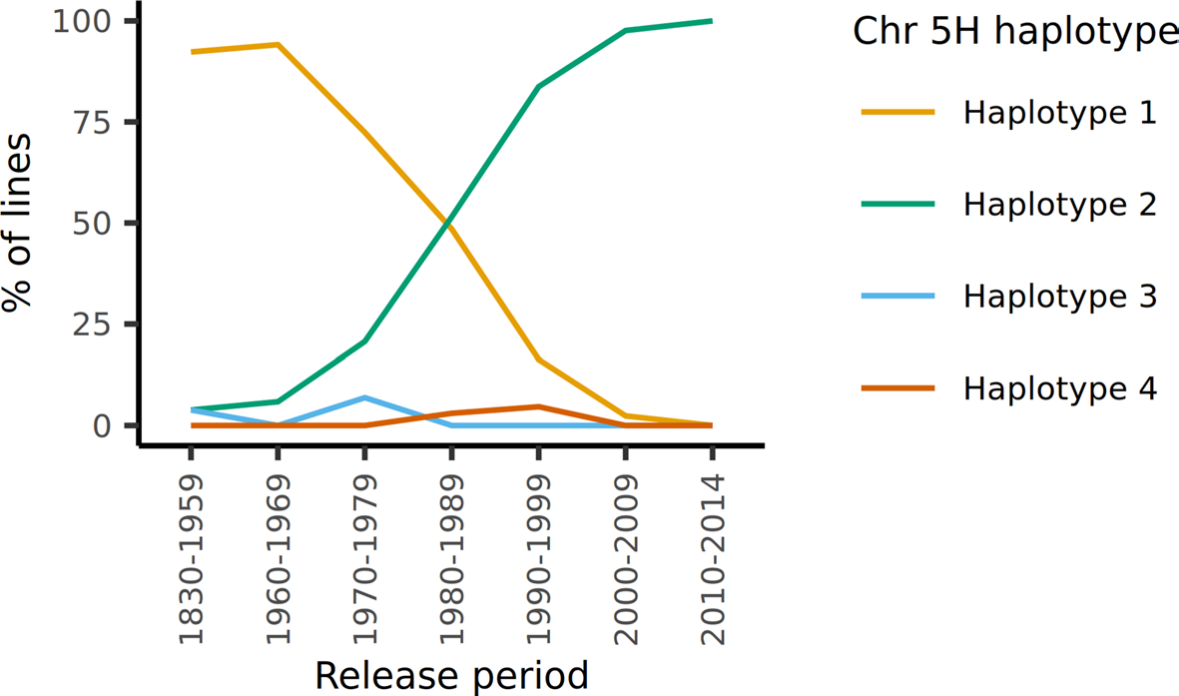
Changes of chromosome 5H haplotype frequencies in the population over time. Colored lines indicate the percentage of cultivars released in a certain time period carrying the respective haplotype (color figure online)

**Fig. 9:**
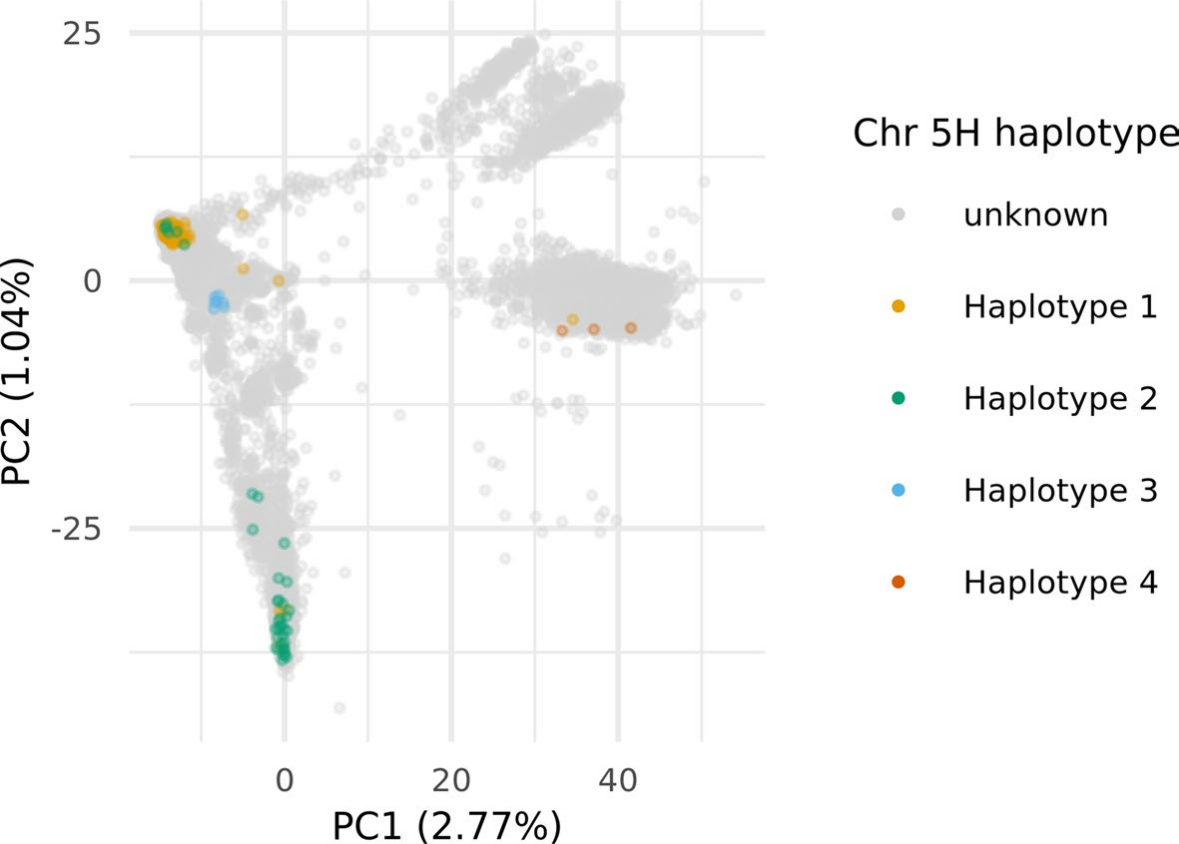
PCoA of all domesticated barley accessions of the German Federal ex situ gene bank at IPK Gatersleben utilizing 6535 SNPs in the haplotype region on chromosome 5H. Colored dots represent accessions carrying the same names as cultivars and landraces from the European two-rowed spring barley panel, color-coded by haplotype. Grey dots represent gene bank accessions not present in the panel (color figure online)

The oldest cultivar in the panel carrying haplotype 2 is ‘Union’, a variety released in 1955 and with southern German pedigree which can be traced back to Bavarian landraces and landrace selections. Over the following 40 years, haplotype 2 quickly increased in frequency in the panel. All but one cultivar released after 2000 carry this haplotype (Fig. 8, Online Resource 1). We utilized the barley collection of the German Federal ex situ gene bank at IPK Gatersleben to identify the haplotypes of accessions in the pedigree of the cultivars in our panel and to make inferences about the origin and geographic distribution of haplotype 2 in a global context (Milner et al. 2019). Eighty-three of the 209 cultivars in our panel are part of the gene bank collection. Including duplicate entries, we retrieved 243 gene bank accessions that carried the same name as cultivars in our panel and that had matching passport data (country of origin, row type and growth habit) (Online Resources 21 and 22). As expected, these accessions formed distinct clusters according to their haplotypes (Fig. 9a). A few accessions known to carry haplotype 1 were apparent in the haplotype 2 cluster and vice versa, which was likely due to genetically distinct accessions carrying the same name and/or incorrect passport information. The k-means cluster analysis did not clearly identify an optimal number of subpopulations. Due to the relatively low number of SNP markers (*n* = 6535) the analysis was not powerful enough to consistently distinguish between haplotypes 1 and 3 and putative additional haplotypes at different values for *k*. However, at *k* >  = 7 the cluster corresponding to haplotype 2 remained stable, hence we assumed *k* = 7 subpopulations (Online Resource 23).

**Fig. 10:**
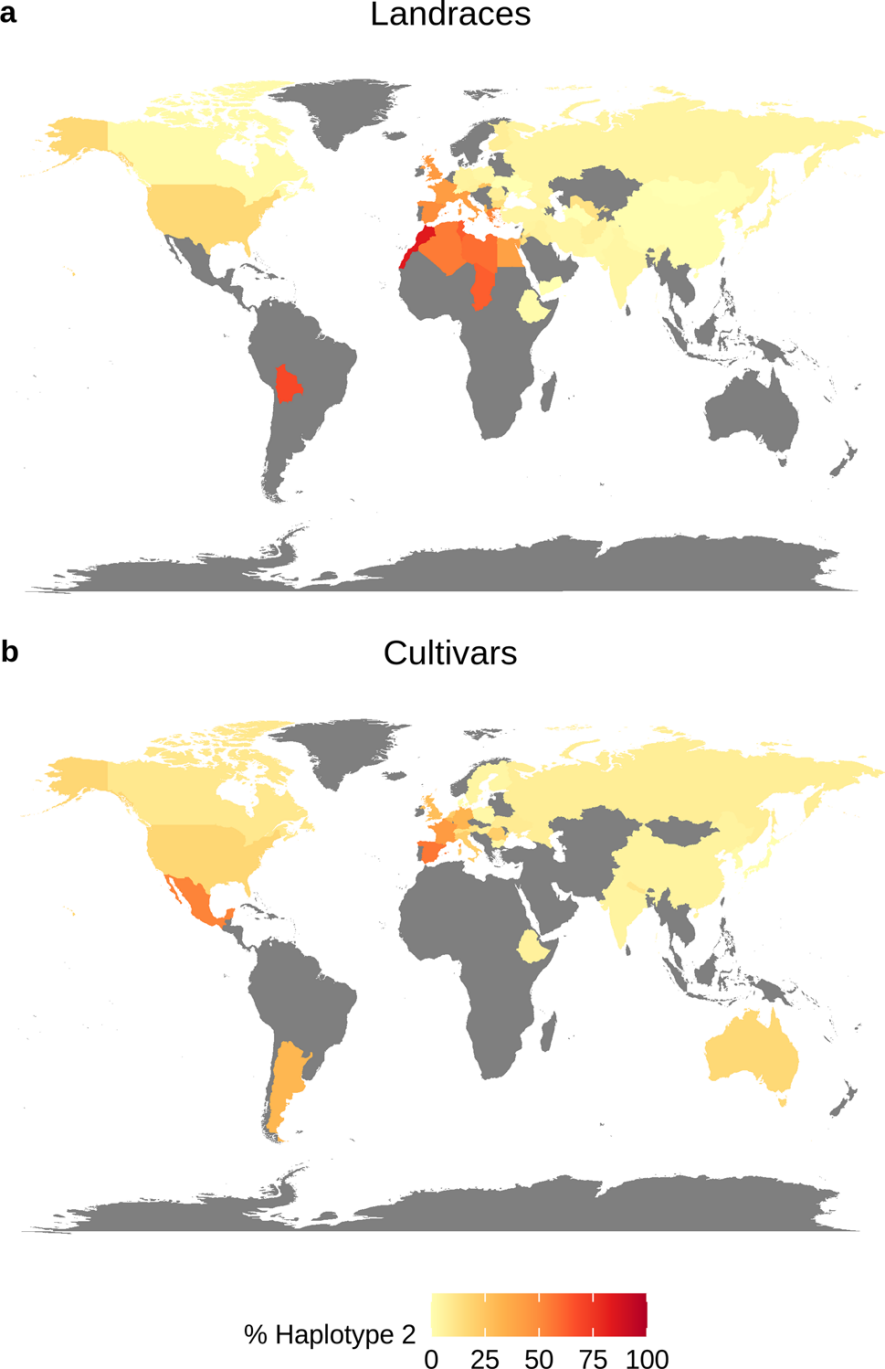
Geographical distribution of haplotype 2 in** a** landraces and** b** cultivars in the IPK gene bank collection. Colors indicate the frequency of gene bank accessions from a given country carrying haplotype 2. Countries from which less than 15 accessions per respective category are present in the gene bank are shown in grey

Northern Africa became apparent as the geographic region with the highest percentage of landraces carrying haplotype 2 (Morocco 84.2%, Chad 63.1% Tunisia, 63.5%, Libya 58.9%, Algeria 56.3%), suggesting this region as the potential origin of this haplotype (Fig. 10a, Online Resource 24). In Europe, haplotype 2 was predominantly found in landraces originating from the Mediterranean region but also from Western Europe as well as the UK. A high frequency of haplotype 2 was also observed among Bolivian and Uruguayan landraces (69.2 and 58.3%, respectively). A north–south gradient of haplotype 2 frequency was apparent in European cultivars (Fig. 10b, Online Resource 24). Fifty-six percent of Spanish cultivars carried haplotype 2. In addition, haplotype 2 was widespread in Mexican cultivars (53.8%). In general, haplotype 2 occurred in higher frequencies among six-rowed and winter cultivars than among two-rowed and spring cultivars, underlining that this haplotype is not row type- or growth habit-specific.

## Discussion

The European two-rowed barley germplasm analysed in this study did not separate into distinct clusters. A general differentiation according to the age of the varieties was apparent which likely reflects the breeding history of the population over time. This differentiation accounted for 16.7% of the genetic variation which is consistent with Tondelli et al. (2013) and Ovesná et al. (2013) who attributed 14% and 23% of the genetic variation to changes over time in European two-rowed spring barleys and Czech barleys, respectively. The DAPC analysis suggested a slight increase in genetic diversity in modern accessions after 1980. The reason for this may be introgressions of exotic germplasm, mainly as a source of disease resistance (Fischbeck 2003). The lack of strong clustering in the population is not surprising, given that most of the modern cultivars can be traced back to founder genotypes that were widely used in European breeding, such as ‘Kenia’, ‘Triumph’, ‘Diamant’, ‘Gull’ or ‘Binder’.

### A major haplotype switch has occurred on chromosome 5H since the 1960s and 1970s

The most striking genetic change over time was observed on chromosome 5H, where a large haplotype block of ~ 250 Mbp was replaced by another haplotype over the course of the last 60 years. This region has been described previously as divergent between different European barley subpopulations. Tondelli et al. (2013) identified high FST values in the pericentromeric region on chromosome 5H between two clusters representing old and new European two-rowed spring barleys. Similarly, the pericentromeric region on chromosome 5H was characterized by high FST values between Polish cultivars released before 1945 and after 2000, respectively (Dziurdziak et al. 2022). Both authors reported low PIC values in the older group and higher PIC values in the newer group of lines studied. This is affected by the composition of the different clusters, i.e. the frequency of the alleles representing the two haplotypes in the clusters and is dependent on the relative impact that the diversity at this locus has on the clustering of the entire population. In our population, both clusters showed low PIC values in this region, suggesting that the division into subpopulations was mostly driven by the genetic differentiation in this region, underlining the importance of this genomic region for genetic differentiation of the whole population.

Using high-density SNP data and gene expression data we are now able to delineate this region more accurately and characterize it more comprehensively. The region between 68.78 and 320.04 Mbp is characterized by high FST values (> 0.7) which differ clearly from the adjacent regions with lower FST values. FST values remain fairly high along the entire region but are especially high at the start and the end of the region. The start and end points of this region are very conserved except in a few cultivars. We did not see any signs of recombination between the two haplotypes in modern European cultivars, which is not uncommon for centromeric and pericentromeric regions of barley chromosomes (Mayer et al. 2012; The International Barley Genome Sequencing Consortium 2012; Mascher et al. 2017). This region showed segregation bias in three out of 45 systematic crosses between 23 spring barleys, making it one of the large regions in which segregation distortion occurred most frequently in a study by Casale et al. (2022). Structural variation, mainly 5–7 Mbp insertions in cultivars carrying haplotype 2, was identified on either side of the haplotype block which may have further reduced collinearity of the two haplotypes and therefore reduced the ability for meiotic recombination. In Arabidopsis, structural rearrangements frequently occur in centromeric and pericentromeric regions (Jiao and Schneeberger 2020). Whereas the haplotype block itself is collinear except for a small inversion at 210 Mbp and a larger inversion around 250 Mbp, the rearrangements in the flanking regions may have had a detrimental effect on recombination and may have thus facilitated the divergence of the two haplotypes.

Within the short time span of approximately 60 years, haplotype 2 has completely replaced the older haplotype 1 in our population which strongly suggests that this region harbors one or more loci associated with beneficial traits which have been under strong selection. We identified 46 genes across the region which were consistently upregulated in all six tissues in haplotype 2 compared to haplotype 1 and two genes which were downregulated in haplotype 2. The annotation of these genes did not suggest an involvement in specific pathways or traits (Online Resources 8 and 20). One of the 46 differentially expressed genes is annotated as a disease resistance protein (CC-NBS-LRR class, BaRT2v18chr5HG236990) family gene (Online Resource 8). Tondelli et al. (2013) and Dziurdzak et al. (2022) both suggested that the leaf rust resistance locus *Rph2* might be the reason for selection of the new haplotype (Borovkova et al. 1997). *Rph2* may thus have conferred resistance to isolates in the North African countries, which led to an increase in haplotype 2 frequency in the North African landraces. Indeed, *Rph2* is one of the most common leaf rust resistance genes in North African barleys (Elmansour et al. 2017). Reinhold and Sharp (1982) showed that cultivars carrying *Rph2* were resistant against some Moroccan isolates but susceptible to others. *Rph2* was originally identified in mostly South American and Australian cultivars such as ‘Weider’, ‘Ricardo’, ‘Peruvian’, ‘Bolivia’, ‘Juliaca’, ‘Quinn’, ‘Purple Nepal’, ‘Modia’, ‘Marco’, ‘Reka I’ and ‘Morocco’ (Henderson 1945; Franckowiak et al. 1997; Sandhu et al. 2012). Today, resistance conferred by *Rph2* has been overcome in many parts of the world including Europe, the US, North Africa, Australia and the Middle East (Reinhold and Sharp 1982; Niks et al. 2000; Mehnaz et al. 2021).

Few virulence tests have been conducted on the cultivars that were part of this study. Among these, ‘Armelle’, ‘Claret’, ‘Delta’, ‘Egmont’, ‘Favorit’, ‘Hart’, ‘Koral’, ‘Krystal’, ‘Rainbow’, ‘Rapid’, ‘Spartan’, ‘Union’ and ‘Zephyr’ were postulated to have Rph2 conferring seedling resistance, whereas ‘Abacus’, ‘Atlas’, ‘Blenheim’, ‘Bonus’, ‘Corniche’, ‘Derkado’, ‘Georgie’, ‘Landlord’, ‘Natasha’, ‘Optic’, ‘Orbit’, ‘Tyne’, ‘Vada’ and ‘Wisa’ were postulated to have other resistance genes but not *Rph2* (Parlevliet 1983; Dreiseitl and Steffenson 2000; Golegaonkar et al. 2009). While this classification is generally in good agreement with the haplotypes present in these cultivars, in a few cultivars the postulated presence or absence of *Rph2* did not coincide with the respective haplotype. ‘Delta’ and ‘Zephyr’ were postulated to carry *Rph2* resistance but were found to have haplotype 1, whereas ‘Blenheim’, ‘Landlord’ and ‘Optic’ were postulated to not carry *Rph2* but do carry haplotype 2. In addition, ‘Abacus’, ‘Georgie’ and ‘Vada’ do not carry *Rph2* but carry the third haplotype likely derived from *H. laevigatum* through ‘Vada’. *H. laevigatum* carries the *Rph20* resistance locus on chromosome 5H which, in contrast to *Rph2*, confers adult plant resistance to *P. hordei*. *Rph20*, however, is located at ~ 7 Mbp on the short arm of chromosome 5H and is therefore not a potential target of selection of haplotype 2. The expression of the NBS-LRR gene BaRT2v18chr5HG236990 and the genotype of SNP S5H_281577407 located within the gene correlate perfectly with the haplotypes we identified; hence, this gene is not a candidate for *Rph2*. We did not identify any SNPs whose genotypes correspond perfectly with the postulated presence or absence of Rhp2. This could be because it was suggested that there are more than two alleles of Rph2, while our dataset contains only biallelic SNPs and will therefore miss more diverse loci. The general good agreement between the presence of *Rph2* and haplotype 2 is not a conclusive proof for *Rph2* being the reason for selection of haplotype 2 but it provides enough evidence to warrant further studies. Ideally, an extensive screening of European and North African barley with isolates from North Africa and Europe representative of different time periods would shed light on the development of virulence over time.

Another potential reason for genetic differentiation is suggested by Contreras-Moreira et al. (2019) who found that the region of 71–348 Mbp (first version of the reference genome of ‘Morex’, Mascher et al. 2017) on chromosome 5H differentiated a group of Spanish six-rowed landraces from coastal regions of Southern Spain from other landraces and cultivars of the Spanish Barley Core Collection (SBCC). The coastal landraces carried a unique haplotype not found in the rest of the SBCC. In addition, this region was associated with agroclimatic variables related to the onset and duration of frost periods as well as vernalization potential. Lower frost tolerance is, however, an unlikely selection target for European barleys and does not explain why this haplotype has increased in frequency so quickly. It is more likely that the lower frost tolerance is linked with another unknown beneficial trait and has therefore been indirectly selected for. In an earlier characterization of the SBCC in terms of agronomic traits, it was shown that this group of landraces had significantly higher leaf rust resistance and higher grain yield under low-yielding conditions than the rest of the collection and was heading earlier than other landraces (Yahiaoui et al. 2014). It is however not clear whether grain yield and earliness are associated with the region on chromosome 5H in the SBCC. Fang et al. (2014) found high levels of linkage disequilibrium, a low number of haplotypes and FST values of > 0.8 in the pericentromeric region of chromosome 5H between wild barleys from the Western and the Eastern part of the Fertile Crescent. This region of high FST was associated with environmental variables related to precipitation. The Western group, which was collected between the Eastern Mediterranean and the Zagros Mountains, originates from a more humid climate than the Eastern group. The authors suggested that positive selection and/or structural rearrangements or suppression of recombination might have caused the differentiation in this region. While the two studies did not conclusively show that adaptation to climatic conditions is the reason for the genetic differentiation between groups in this genomic region, it is worthwhile to test this in further studies.

The haplotype frequency screening of the IPK gene bank identified Northern Africa as the geographic region with the highest proportion of accessions carrying haplotype 2. It is therefore likely that the haplotype first occurred in this region and that the haplotype confers a selective advantage under Northern African conditions. However, care must be taken when making inferences based on the gene bank data alone. While being one of the largest barley germplasm collections worldwide, it is not free from biases. For example, modern cultivars and breeding material are not well-represented in the collection due to proprietary reasons, so the frequencies of haplotype 2 shown in Fig. 10 are likely an underestimation as they do not reflect the strong selection for this haplotype in Europe in the last decades that we have seen in our panel. Furthermore, as many countries are underrepresented in the collection, passport data of many accessions are incomplete, and different collections may be subject to different sampling biases, the global haplotype distribution is equally incomplete and needs to be interpreted with caution.

Similarly, potential mismatches between gene bank genotypes and passport data became apparent when we attempted to identify the haplotypes of accessions that were progenies of the oldest carriers of haplotype 2 in our panel. The oldest accession in the panel, Union, is a cross between a cross of ‘Weihenstephaner Mehltauresistente II’ x ‘Donaria’ and ‘Firlbeck 621’. All accessions containing ‘Mehltauresistente’ (= resistant to powdery mildew) in their name were assigned to the haplotype 1 cluster, excluding this parent as the likely haplotype donor for ‘Union’. Two out of three accessions named ‘Donaria’ were part of the haplotype 1 cluster while the third one belonged to another group. The collection contains one accession called ‘Firlbecks III/621’ and two accessions called ‘Firlbecks III Neu’. The former possesses haplotype 2 while the latter two carry haplotype 1. It is therefore not possible to conclude that ‘Firlbeck 621’ is the haplotype 2 donor of ‘Union’. While ‘Firlbecks Union’, ‘UNION (BENDER) AUSGANGSFORM 4X’ and two accessions called ‘Union’ all have haplotype 2, there is also one accession named ‘UNION AUSGANGSFORM 2X’ which carries haplotype 1. ‘Firlbeck III’ is derived from a cross between ‘Firlbeck II’ and ‘Haisa’. ‘Firlbeck II’ is not part of the genebank collection and none of the accessions including the name ‘Haisa’ had haplotype 2 except for one accession simply named ‘Haisa’. ‘Haisa’ is likely ‘Haisa I’, a cross between ‘Heines Hanna’ and ‘Ackermann Isaria’. None of the accessions containing the names ‘Hanna’ and ‘Isaria’ carried haplotype 2. Despite these limitations of utilizing passport data and genetic information from gene bank collections we do believe, however, that the general patterns of haplotype distribution are reliable, simply due to the large number of accessions analysed.

### Implications for barley breeding

The presence of a 250 Mbp genomic region characterized by lack of recombination and high levels of fixation in modern barley germplasm has substantial implications for barley breeding. Contemporary barley breeding relies primarily on the selection of favourable combinations of alleles generated by crosses between breeder strains (Fischbeck 2003). While it could be argued that this haplotype has remained largely unchanged since its introduction in the 1950s because it already represents the best combination of favourable alleles, it appears more likely that the lack of recombination has hampered the selection of better combinations. The region contains 1080 genes (2.7% of the 39,419 genes in BaRTv2.0) and it is very unlikely that they all carry the most beneficial allele, especially since this haplotype has developed in a completely different environment (North Africa, in landraces) compared to where it is “used” now (Europe, intensive agriculture).

Given the high prevalence of haplotype 2 in current European two-rowed spring barleys it is likely that haplotype 2 is also present at very high frequencies in the breeding material of most Central, Western and Southern European breeding programs. Hence, current barley breeding methods are unlikely to be able to increase genetic diversity in this region. Due to the low recombination rate in centromeric and pericentromeric regions of barley chromosomes (Mayer et al. 2012; Baker et al. 2014) crosses between genotypes carrying different haplotypes are not an efficient way of breaking the linkage and increasing diversity in this region. Instead, a more promising approach for increasing genetic variation and exploiting new allelic combinations is to screen for different 5H haplotypes present in landraces and unadapted material (e.g. crop wild relatives) for association with beneficial traits. This approach already proved successful in the past when *H. laevigatum* was widely used as a crossing partner in European barley breeding for its resistance against powdery mildew and leaf rust. A similar approach may be necessary to increase genetic variation in the centromeric regions of chromosomes 1H, 2H and 7H characterized by low diversity in spring barley (Mascher et al. 2017; Schreiber et al. 2023).

## Conclusions

In conclusion, we identified a ~ 250 Mbp centromeric region on chromosome 5H which has been under strong selection in European barley cultivars in the past 70 years. We speculate that the haplotype 2 first occurred in North Africa and conferred a selective advantage over other haplotypes, possibly due to leaf rust resistance. It may then have been introduced to Southern European landraces and thus found its way into European cultivars via this route. Similarly, it may have been introduced to South America via Spanish landraces as well (Friedt et al. 2011). This proposed route is highly speculative and further studies will be required, especially to test the hypothesis of improved stress resistance (diseases, temperatures). To increase genetic variation in this region and break potential linkage between beneficial and detrimental traits we propose to screen and characterize germplasm carrying different haplotypes in this genomic region.

## Supplementary Information

Below is the link to the electronic supplementary material.

**Online Resource 1** List of cultivars in the European two-rowed spring barley panel including country of origin, release year, chromosome 5H haplotype and cluster membership at k=2 and k=3

**Online Resource 2** Pairwise FST between different release periods

**Online Resource 3** Analysis of Molecular Variance (AMOVA) statistics quantifying the extent of genetic variation found between and within groups of cultivars defined by release periods (1830-1959, 1960-1979, 1980-1999, 2000-2014)

**Online Resource 4** Neighbor-joining tree using the genotypes of 1,509,447 SNP markers. The cultivars are color-coded according to the period in which they were released. The inner ring panel shows the chromosome 5H haplotype of each cultivar, the outer ring panels show the k-means cluster membership of each cultivar at k=2 (outermost ring panel) and k=3 (central ring panel)

**Online Resource 5** Principal coordinate analysis of the European two-rowed spring barley panel using the genotypes of 1,509,447 SNP markers. Each dot represents a cultivar, color-coded according to geographic origin

**Online Resource 6** Left: Cumulative percentage of variance explained by different numbers of PCs. Selecting the number of PCs at which ~95% of the cumulative variance were explained, 110 PCs were retained for k-means clustering. Right: Bayesian Information Criterion (BIC) values at different numbers of assumed subpopulations (k). Low BIC values indicate a good fit of the model to the data. Using the elbow method to determine the value for k at which the curve flattens, two or three subpopulations were assumed to be likely

**Online Resource 7** k-means cluster membership of cultivars at k=2 (A) and k=3 (B) superimposed on PCoA (see Fig. 3)

**Online Resource 8** Annotation, gene IDs and genetic positions in reference genomes Barke and Morex V3 as well as the reference transcriptome BaRTv2 of the 48 genes differentially expressed in haplotype 1 and 2. These 48 genes form a distinct group in a PCoA of all genes located between 69 and 320 Mbp on chromosome 5H (see Fig. 7, red clusters). All genes were highly expressed in cultivars carrying haplotype 2 except for the two genes marked with asterisks which were highly expressed in cultivars carrying haplotype 1 and low/not expressed in cultivars carrying haplotype 2

**Online Resource 9** Heatmap of the expression of the genes between 69 and 320 Mbp on chromosome 5H forming a separate k-means cluster and showing low or no expression in cultivars carrying the old haplotype and higher expression in cultivars carrying the new haplotype in the crown tissue of one week old seedlings. An exception is BaRT2v18chr5HG233580 which shows the opposite pattern. Gene expression is not correlated with haplotypes 3 and 4.

**Online Resource 10** Heatmap of the expression of the genes between 69 and 320 Mbp on chromosome 5H forming a separate k-means cluster and showing low or no expression in cultivars carrying the old haplotype and higher expression in cultivars carrying the new haplotype in the root tissue of one week old seedlings. An exception is BaRT2v18chr5HG233580 which shows the opposite pattern. Gene expression is not correlated with haplotypes 3 and 4

**Online Resource 11** Heatmap of the expression of the genes between 69 and 320 Mbp on chromosome 5H forming a separate k-means cluster and showing low or no expression in cultivars carrying the old haplotype and higher expression in cultivars carrying the new haplotype in the developing inflorescence tissue. Exceptions are BaRT2v18chr5HG233570 and BaRT2v18chr5HG233580 which show the opposite pattern. Gene expression is not correlated with haplotypes 3 and 4

**Online Resource 12** Heatmap of the expression of the genes between 69 and 320 Mbp on chromosome 5H forming a separate k-means cluster and showing low or no expression in cultivars carrying the old haplotype and higher expression in cultivars carrying the new haplotype in the peduncle tissue. Exceptions are BaRT2v18chr5HG233570 and BaRT2v18chr5HG233580 which show the opposite pattern. Gene expression is not correlated with haplotypes 3 and 4

**Online Resource 13** Heatmap of the expression of the genes between 69 and 320 Mbp on chromosome 5H forming a separate k-means cluster and showing low or no expression in cultivars carrying the old haplotype and higher expression in cultivars carrying the new haplotype in the spikelet tissue. Exceptions are BaRT2v18chr5HG233570 and BaRT2v18chr5HG233580 which show the opposite pattern. Gene expression is not correlated with haplotypes 3 and 4

**Online Resource 14** Heatmap of the expression of the genes between 69 and 320 Mbp on chromosome 5H forming a separate k-means cluster and showing low or no expression in cultivars carrying the old haplotype and higher expression in cultivars carrying the new haplotype in the developing grain tissue. Gene expression is not correlated with haplotypes 3 and 4

**Online Resource 15** PCA of all cultivars in the panel, using 110,914 SNPs at 68.78 - 320.04 Mbp on chromosome 5H color-coded by the haplotype the cultivars carry in this region

**Online Resource 16** Alleles of 10,000 randomly selected SNP markers in the haplotype region between 68.78 and 320.04 Mbp on chromosome 5H and the flanking 10 Mbp on each side. Red indicates the homozygous reference genotype (cultivar ‘Barke’), yellow indicates the homozygous alternative genotype, green indicates the heterozygous genotype, grey indicates missing data. White columns indicate regions without SNP data. Accessions are sorted according to haplotype (right bar: orange = haplotype 1, green = haplotype 2, blue = haplotype 3, red = haplotype 4)

**Online Resource 17** a) Alleles of all SNP markers 30 Mbp upstream and 4 Mbp downstream of the start of the haploblock region on chromosome 5H (38.78-72.78 Mbp). b) Alleles of all SNP markers 4 Mbp upstream and 30 Mbp downstream of the end of the haploblock region on chromosome 5H (316.04-350.04 Mbp). c) Alleles of all SNP markers 0.5 Mbp upstream and 0.5 Mbp downstream of the start of the haploblock region on chromosome 5H (68.28-69.28 Mbp). b) Alleles of all SNP markers 0.5 Mbp upstream and 0.5 Mbp downstream of the end of the haploblock region on chromosome 5H (319.54-320.54 Mbp). Black lines indicate the start (a, c) and end (b, d) of the haploblock region, respectively. Red indicates the homozygous reference genotype (cultivar ‘Barke’), yellow indicates the homozygous alternative genotype, green indicates the heterozygous genotype, grey indicates missing data. White columns indicate regions without SNP data. Accessions are sorted according to haplotype (right bar: orange = haplotype 1, green = haplotype 2, blue = haplotype 3, red = haplotype 4)

**Online Resource 18** Dotplots showing genome alignments of cv. Morex (carrying haplotype 1) and cv. Barke (carrying haplotype 2) of a) the entire haplotype region on chromosome 5H illustrating the structural variation at the start and end of the region, b) the start of the haplotype region illustrating an insertion in Barke followed by two inversions further downstream and c) the end of the haplotype region illustrating an insertion in Barke followed by a deletion or potential translocation

**Online Resource 19** Dotplots showing pairwise alignments of the chromosome 5H haplotypes in genomes of nine cultivars/accessions carrying haplotype 1 or 2. Names of the cultivars/accessions are shown on the bottom and left. Accessions carrying haplotype 1 are ‘Golden Promise’, ‘Morex’, ‘HOR13821’, ‘HOR3081’ and ‘HOR3365’. Accessions carrying haplotype 2 are ‘Barke, ‘Igri’, ‘HOR13942’ and ‘RGT Planet’.

**Online Resource 20** GO enrichment of the 1080 genes located between 68.78 and 320.04 Mbp on chromosome 5H. Shown are the GO IDs, GO terms, the number of annotated genes per term in BartV2.0, the number of genes observed per term, the number of genes expected per term, the p-value of gene enrichment and the GO category

**Online Resource 21** PCAs of all domesticated barley accessions of the German Federal ex-situ gene bank at IPK Gatersleben. Red dots represent the 243 accessions carrying the same names as cultivars and landraces from the European two-rowed spring barley panel. The figure was created using the IPK Bridge Portal (IPK Gatersleben - BRIDGE Web Portal (ipk-gatersleben.de)

**Online Resource 22** PCoAs of all domesticated barley accessions of the German Federal ex-situ gene bank at IPK Gatersleben. Accessions are color-coded by geographic origin. NZ=New Zealand

**Online Resource 23** Clustering of domesticated barley accessions of the IPK gene bank at different k. While the approach is not suitable to clearly distinguish haplotype 1, haplotype 3 and other potential haplotypes clustering together, haplotype 2 is clearly distinguished at k >= 7

**Online Resource 24** Geographical distribution of haplotype 2 in groups of landraces and cultivars characterized by different row-types and growth habits in the IPK gene bank collection. Colors indicate the frequency of gene bank accessions from a given country carrying haplotype 2. Countries from which less than 15 accessions per respective category are present in the gene bank are shown in grey

## Data Availability

This study is based on datasets which are described in detail in Schreiber et al. (2023). Raw data files for both RNA-sequence data and whole genome shotgun data have been deposited at the European Nucleotide Archive (ENA) under the project numbers PRJEB49069 and PRJEB48903, respectively. SNP data is available through the Germinate platform https://ics.hutton.ac.uk/germinate-barn/. Gene expression data is available on the e!DAL platform under the preview link https://doi.ipk-gatersleben.de/DOI/815787d8-4036-408b-999e-725f7645eacf/6e532074-b4d7-4393-9221-8fe643e100f2/2/1847940088. Code used in this study is available at https://github.com/rwonneberger/Wonneberger_Chr5H_haplotype_barley.
