# Supplementary material for: Major chromosome 5H haplotype switch structures the European two-rowed spring barley germplasm of the past 190 years
Source: Theor Appl Genet. 2023 Jul 21;136(8):174. doi: 10.1007/s00122-023-04418-7 (PMC10361897; doi:10.1007/s00122-023-04418-7)
Supplement: Supplementary file 2 — Online Resource 2 Pairwise FST between different release periods [file 122_2023_4418_MOESM2_ESM.docx]

**Major chromosome 5H haplotype switch structures the European two-rowed spring barley germplasm of the past 190 years**

Ronja Wonneberger, Miriam Schreiber, Allison Haaning, Gary J. Muehlbauer, Robbie Waugh, Nils Stein (stein@ipk-gatersleben.de)

Theoretical and Applied Genetics

**Online Resource 2** Pairwise FST between different release periods

| FST | 1830-1959 | 1960-1979 | 1980-1999 | 2000-2014 |
| --- | --- | --- | --- | --- |
| 1960-1979 | 0.010 | 0 |  |  |
| 1980-1999 | 0.147 | 0.104 | 0 |  |
| 2000-2014 | 0.303 | 0.258 | 0.058 | 0 |
