# Supplementary material for: Major chromosome 5H haplotype switch structures the European two-rowed spring barley germplasm of the past 190 years
Source: Theor Appl Genet. 2023 Jul 21;136(8):174. doi: 10.1007/s00122-023-04418-7 (PMC10361897; doi:10.1007/s00122-023-04418-7)
Supplement: Supplementary file 3 — Online Resource 3 Analysis of Molecular Variance (AMOVA) statistics quantifying the extent of genetic variation found between and within groups of cultivars defined by release periods (1830-1959, 1960-1979, 1980-1999, 2000-2014) [file 122_2023_4418_MOESM3_ESM.docx]

**Major chromosome 5H haplotype switch structures the European two-rowed spring barley germplasm of the past 190 years**

Ronja Wonneberger, Miriam Schreiber, Allison Haaning, Gary J. Muehlbauer, Robbie Waugh, Nils Stein (stein@ipk-gatersleben.de)

Theoretical and Applied Genetics

**Online Resource 3** Analysis of Molecular Variance (AMOVA) statistics quantifying the extent of genetic variation found between and within groups of cultivars defined by release periods (1830-1959, 1960-1979, 1980-1999, 2000-2014)

| Source | Df ^a^ | SS ^b^ | MS ^c^ | Estimated variance | Percentage % | p value |
| --- | --- | --- | --- | --- | --- | --- |
| Between release period | 3 | 4539736 | 1513245 | 18612 | 14.0 | 0.001 |
| Within release period | 197 | 33898870 | 172075 | 114251 | 86.0 | 0.001 |
| Total | 200 | 38438606 | 192193 | 135512 |  |  |

^a^ Degrees of freedom, ^b^ Sum of squares, ^c^ Mean square
