# Supplementary figures and images for: Major chromosome 5H haplotype switch structures the European two-rowed spring barley germplasm of the past 190 years
Source: Theor Appl Genet. 2023 Jul 21;136(8):174. doi: 10.1007/s00122-023-04418-7 (PMC10361897; doi:10.1007/s00122-023-04418-7)

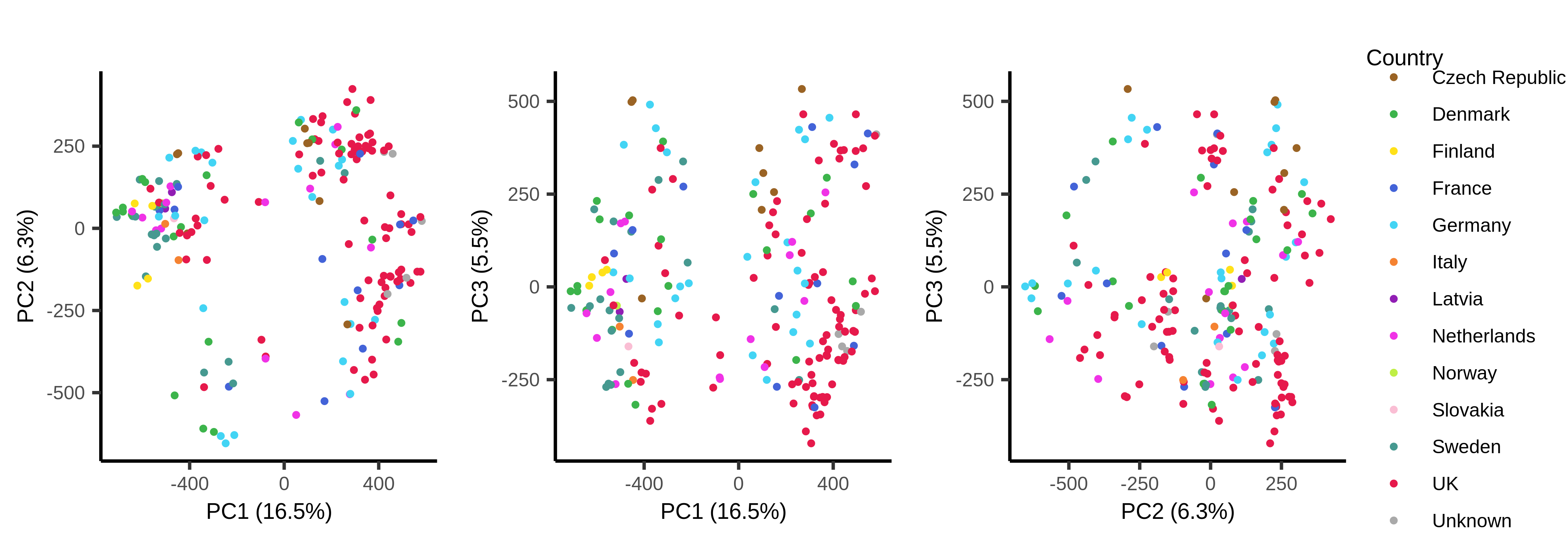

Supplement: Supplementary file 5 — Online Resource 5 Principal coordinate analysis of the European two-rowed spring barley panel using the genotypes of 1,509,447 SNP markers. Each dot represents a cultivar, color-coded according to geographic origin [file 122_2023_4418_MOESM5_ESM.tiff]

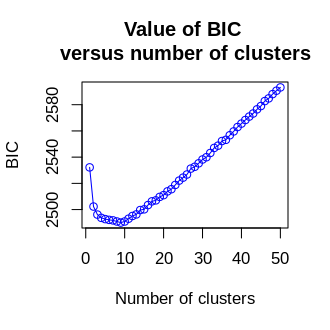

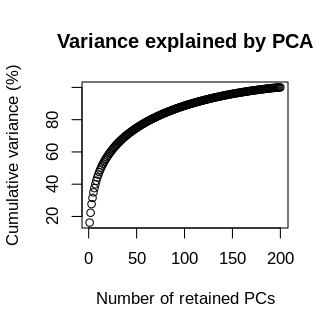

Supplement: Supplementary file 6 — Online Resource 6 Left: Cumulative percentage of variance explained by different numbers of PCs. Selecting the number of PCs at which ~95% of the cumulative variance were explained, 110 PCs were retained for k-means clustering. Right: Bayesian Information Criterion (BIC) values at different numbers of assumed subpopulations (k). Low BIC values indicate a good fit of the model to the data. Using the elbow method to determine the value for k at which the curve flattens, two or three subpopulations were assumed to be likely [file 122_2023_4418_MOESM6_ESM.docx]

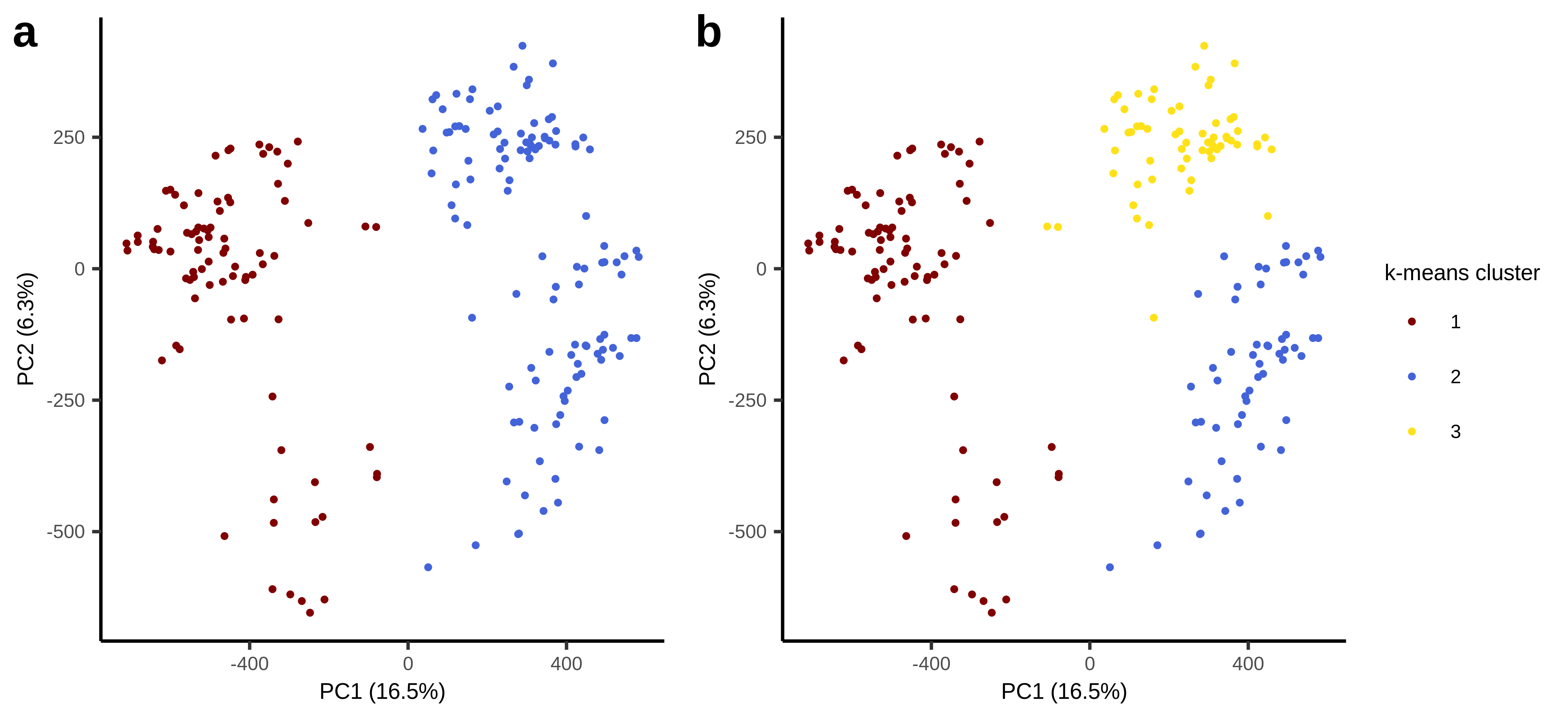

Supplement: Supplementary file 7 — Online Resource 7 k-means cluster membership of cultivars at k=2 (A) and k=3 (B) superimposed on PCoA (see Fig. 3) [file 122_2023_4418_MOESM7_ESM.tiff]

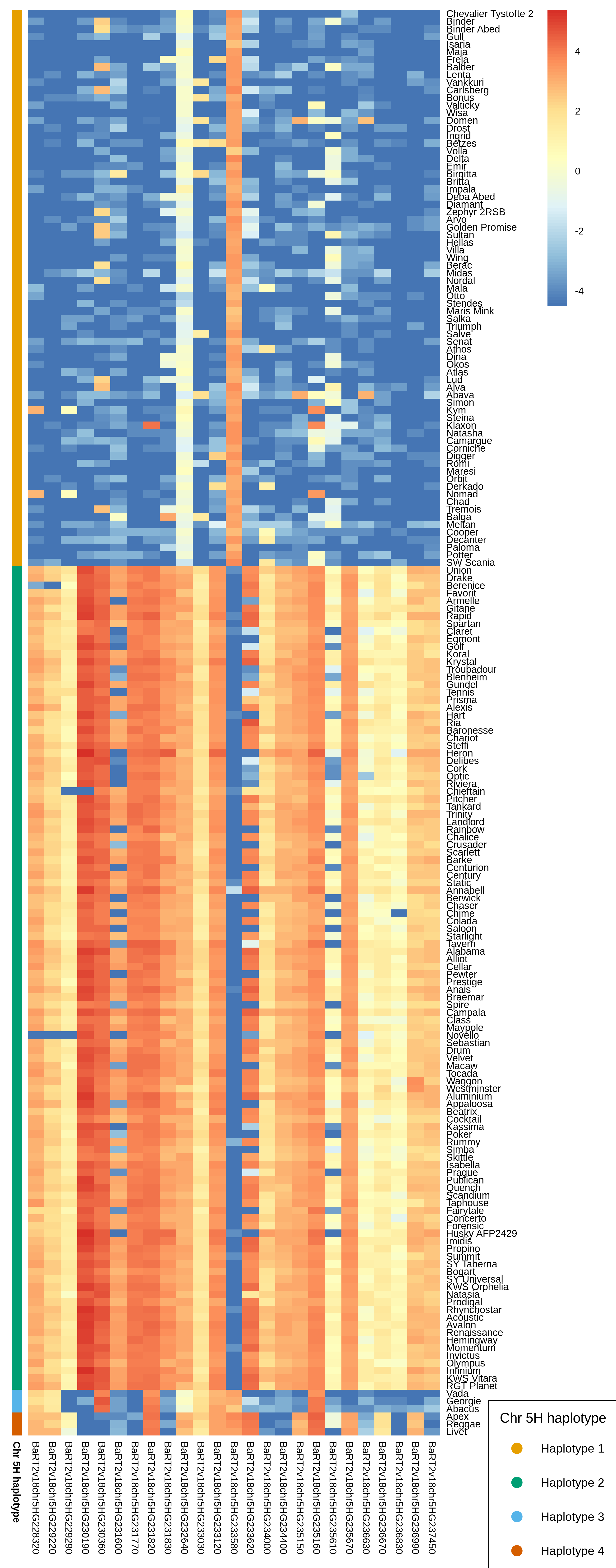

Supplement: Supplementary file 9 — Online Resource 9 Heatmap of the expression of the genes between 69 and 320 Mbp on chromosome 5H forming a separate k-means cluster and showing low or no expression in cultivars carrying the old haplotype and higher expression in cultivars carrying the new haplotype in the crown tissue of one week old seedlings. An exception is BaRT2v18chr5HG233580 which shows the opposite pattern. Gene expression is not correlated with haplotypes 3 and 4. [file 122_2023_4418_MOESM9_ESM.tiff]

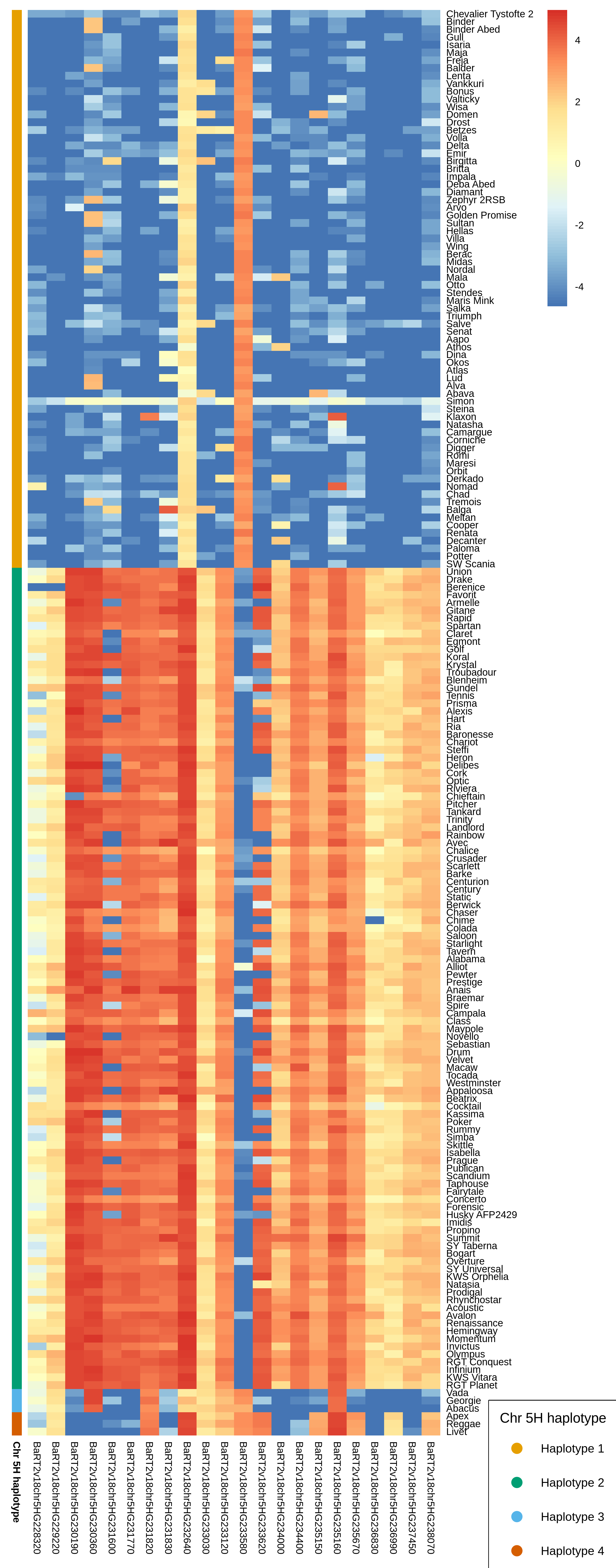

Supplement: Supplementary file 10 — Online Resource 10 Heatmap of the expression of the genes between 69 and 320 Mbp on chromosome 5H forming a separate k-means cluster and showing low or no expression in cultivars carrying the old haplotype and higher expression in cultivars carrying the new haplotype in the root tissue of one week old seedlings. An exception is BaRT2v18chr5HG233580 which shows the opposite pattern. Gene expression is not correlated with haplotypes 3 and 4 [file 122_2023_4418_MOESM10_ESM.tiff]

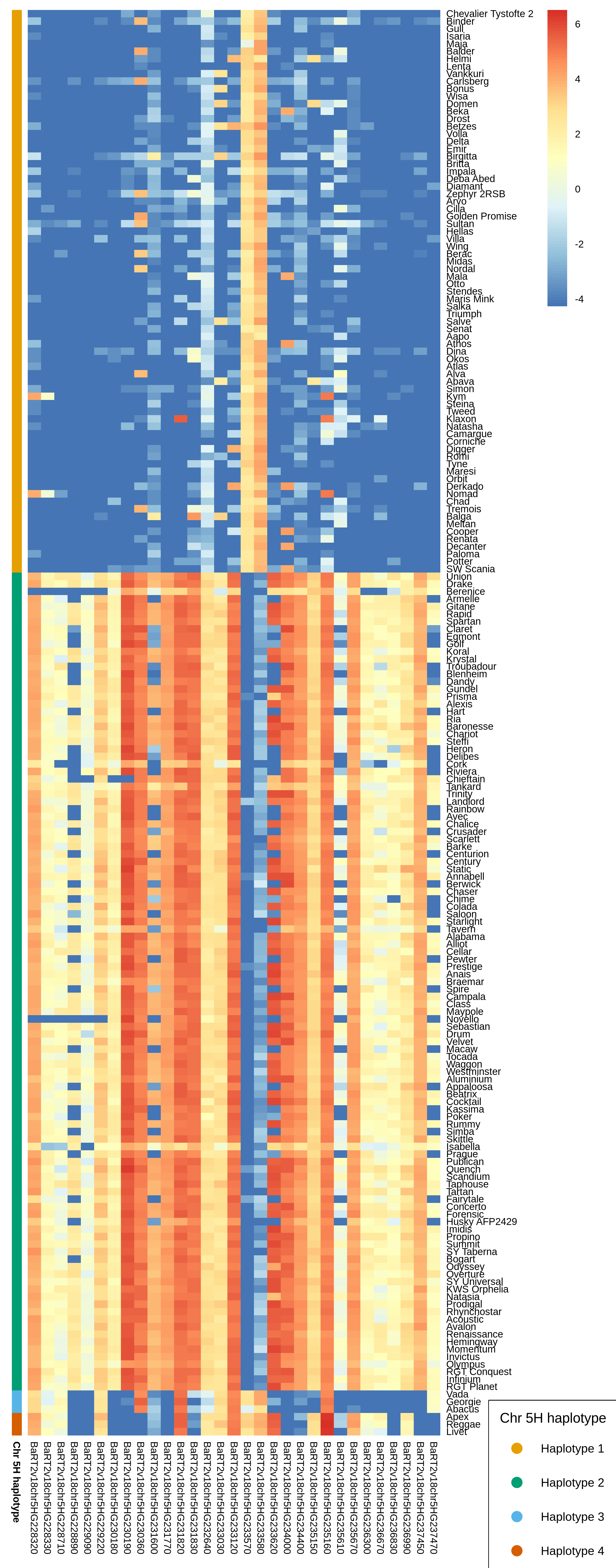

Supplement: Supplementary file 11 — Online Resource 11 Heatmap of the expression of the genes between 69 and 320 Mbp on chromosome 5H forming a separate k-means cluster and showing low or no expression in cultivars carrying the old haplotype and higher expression in cultivars carrying the new haplotype in the developing inflorescence tissue. Exceptions are BaRT2v18chr5HG233570 and BaRT2v18chr5HG233580 which show the opposite pattern. Gene expression is not correlated with haplotypes 3 and 4 [file 122_2023_4418_MOESM11_ESM.tiff]

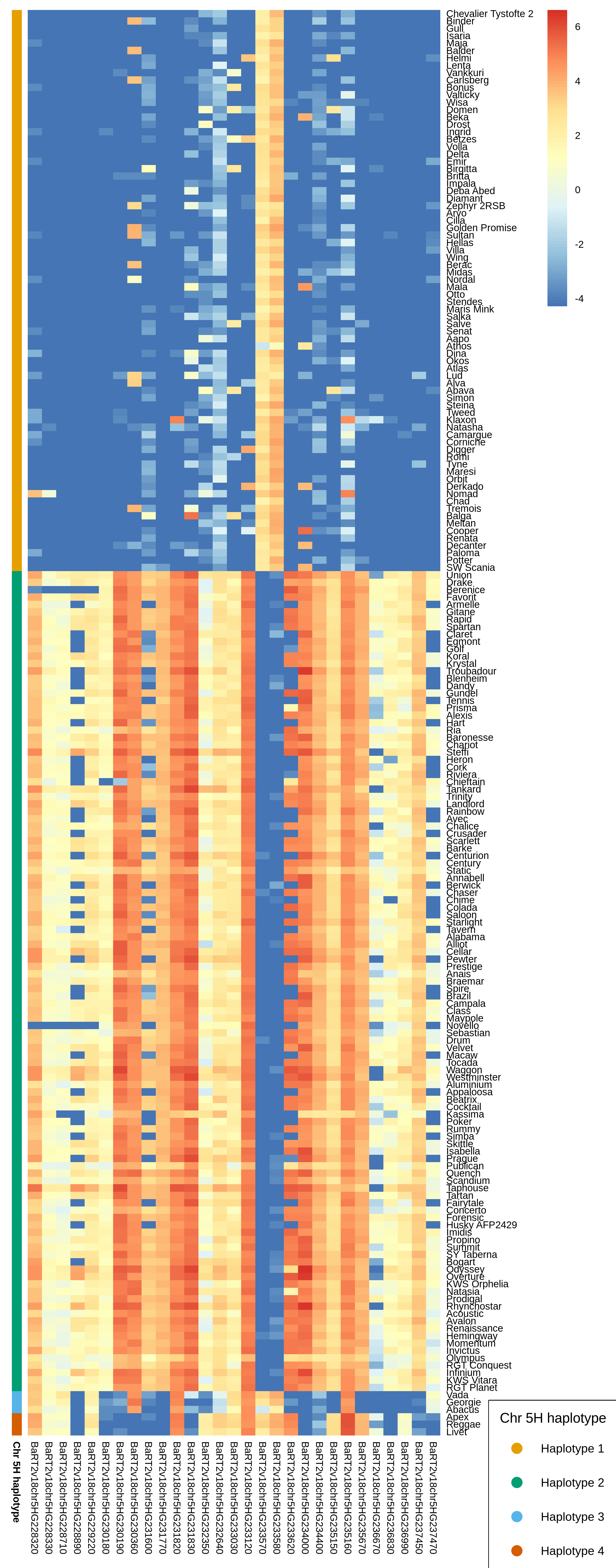

Supplement: Supplementary file 12 — Online Resource 12 Heatmap of the expression of the genes between 69 and 320 Mbp on chromosome 5H forming a separate k-means cluster and showing low or no expression in cultivars carrying the old haplotype and higher expression in cultivars carrying the new haplotype in the peduncle tissue. Exceptions are BaRT2v18chr5HG233570 and BaRT2v18chr5HG233580 which show the opposite pattern. Gene expression is not correlated with haplotypes 3 and 4 [file 122_2023_4418_MOESM12_ESM.tiff]

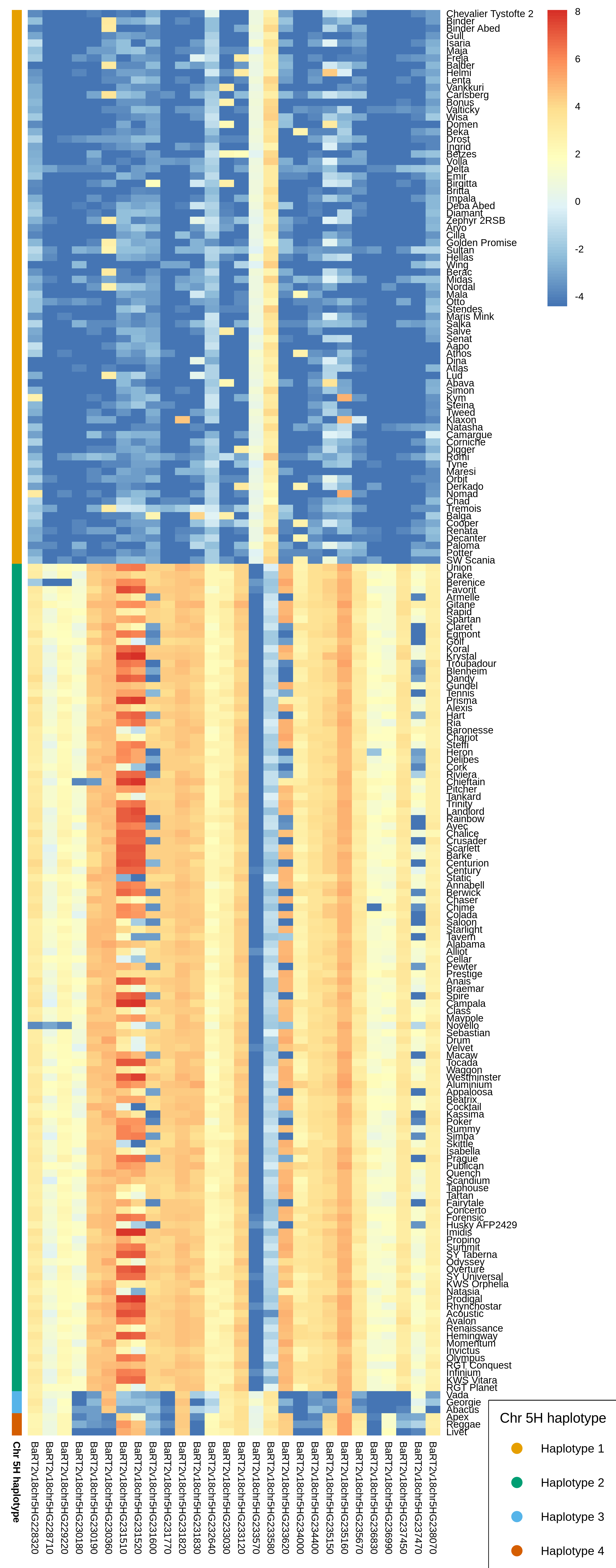

Supplement: Supplementary file 13 — Online Resource 13 Heatmap of the expression of the genes between 69 and 320 Mbp on chromosome 5H forming a separate k-means cluster and showing low or no expression in cultivars carrying the old haplotype and higher expression in cultivars carrying the new haplotype in the spikelet tissue. Exceptions are BaRT2v18chr5HG233570 and BaRT2v18chr5HG233580 which show the opposite pattern. Gene expression is not correlated with haplotypes 3 and 4 [file 122_2023_4418_MOESM13_ESM.tiff]

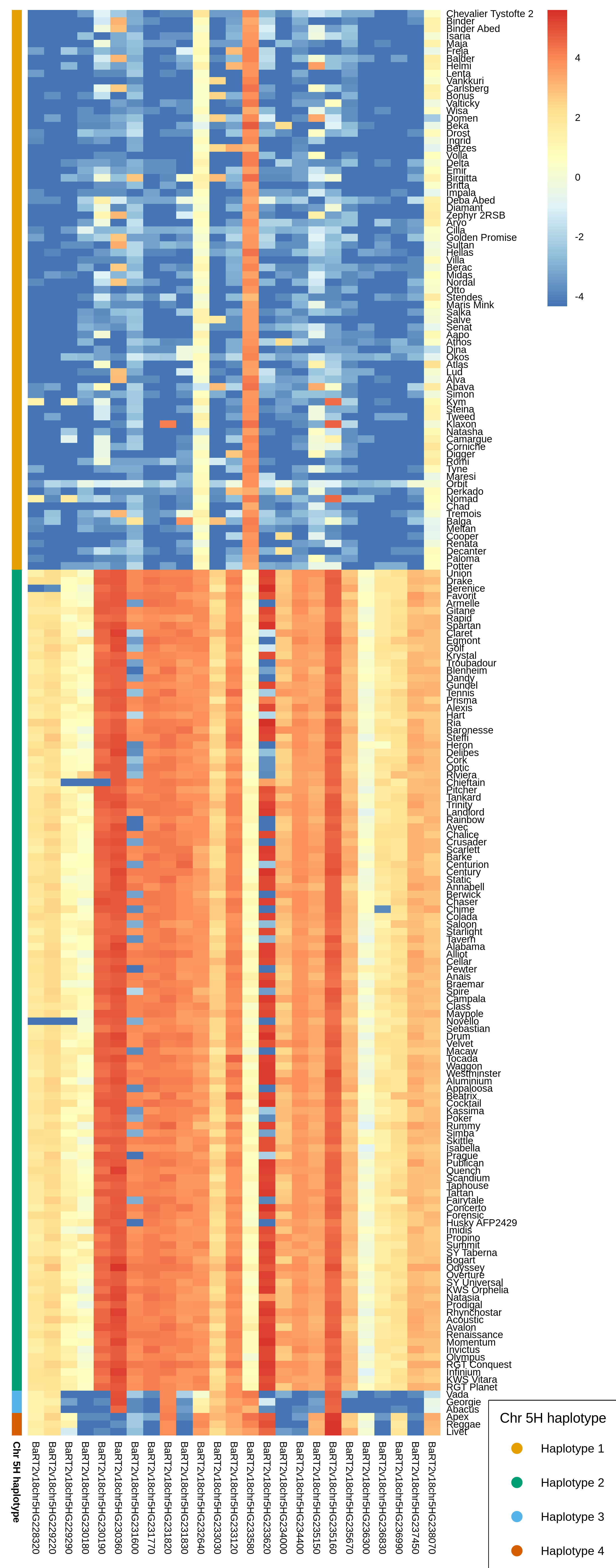

Supplement: Supplementary file 14 — Online Resource 14 Heatmap of the expression of the genes between 69 and 320 Mbp on chromosome 5H forming a separate k-means cluster and showing low or no expression in cultivars carrying the old haplotype and higher expression in cultivars carrying the new haplotype in the developing grain tissue. Gene expression is not correlated with haplotypes 3 and 4 [file 122_2023_4418_MOESM14_ESM.tiff]

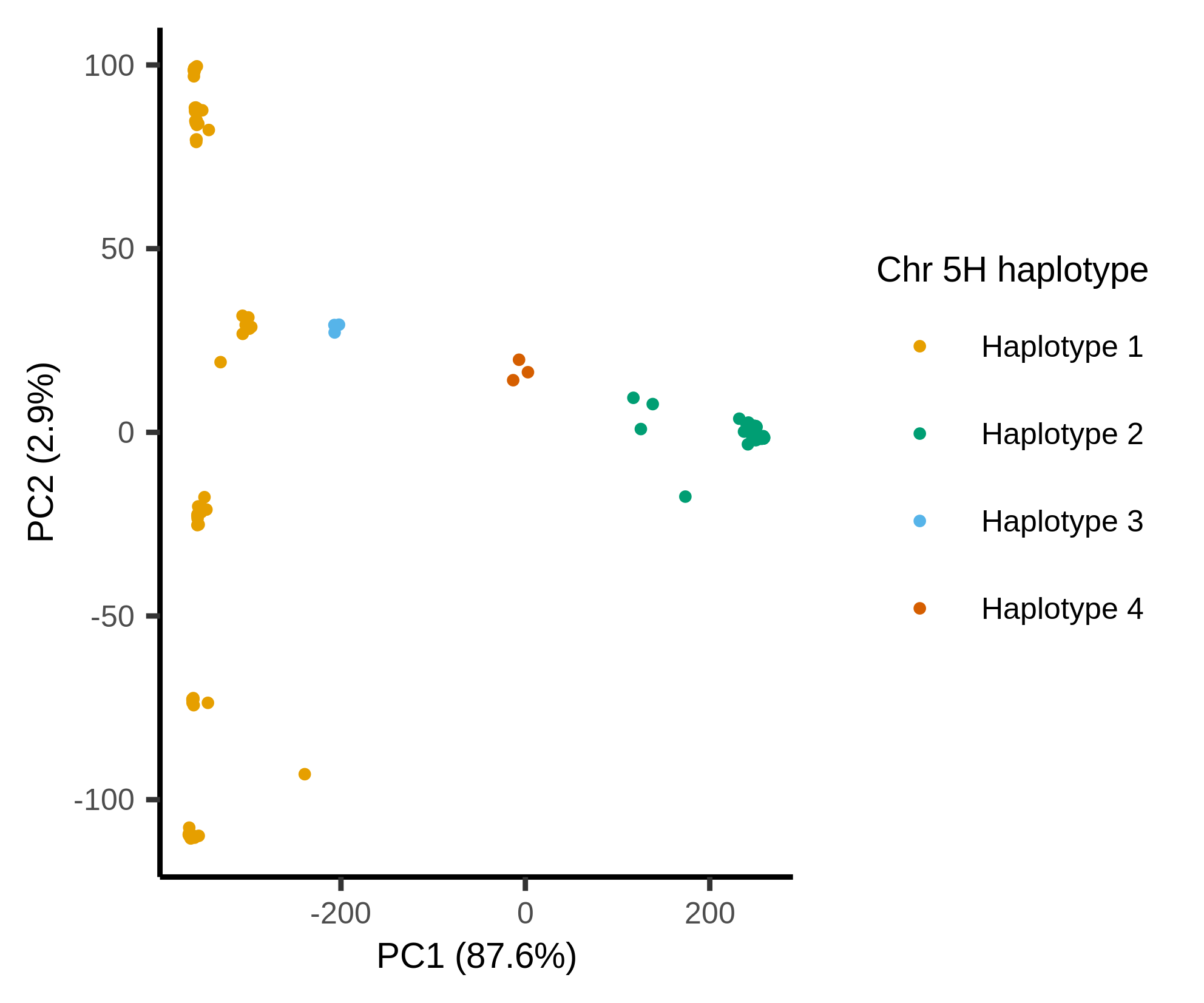

Supplement: Supplementary file 15 — Online Resource 15 PCA of all cultivars in the panel, using 110,914 SNPs at 68.78 - 320.04 Mbp on chromosome 5H color-coded by the haplotype the cultivars carry in this region [file 122_2023_4418_MOESM15_ESM.tiff]

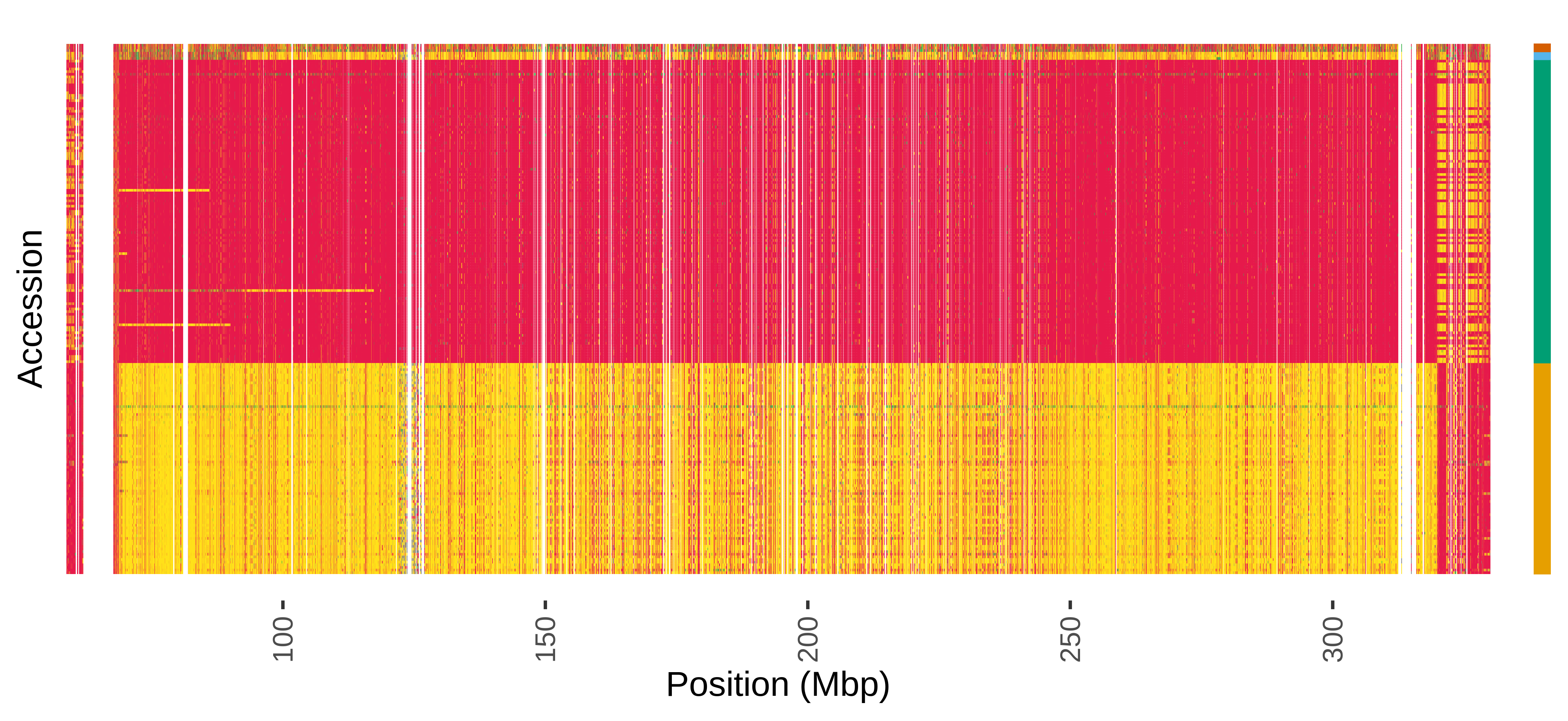

Supplement: Supplementary file 16 — Online Resource 16 Alleles of 10,000 randomly selected SNP markers in the haplotype region between 68.78 and 320.04 Mbp on chromosome 5H and the flanking 10 Mbp on each side. Red indicates the homozygous reference genotype (cultivar ‘Barke’), yellow indicates the homozygous alternative genotype, green indicates the heterozygous genotype, grey indicates missing data. White columns indicate regions without SNP data. Accessions are sorted according to haplotype (right bar: orange = haplotype 1, green = haplotype 2, blue = haplotype 3, red = haplotype 4) [file 122_2023_4418_MOESM16_ESM.tiff]

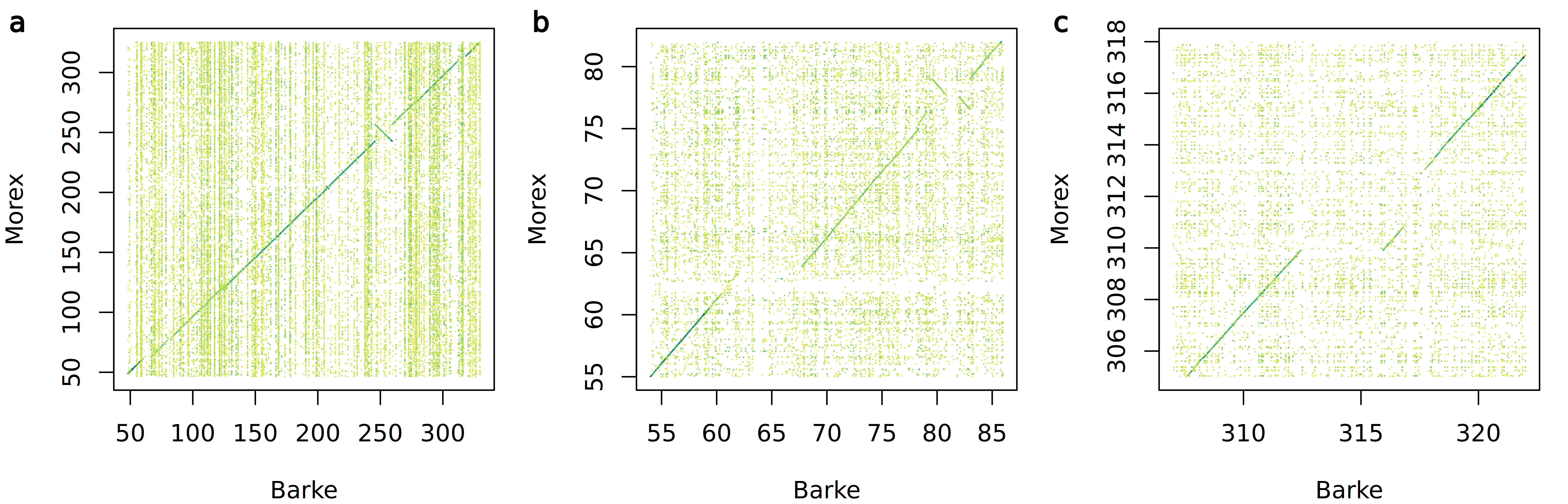

Supplement: Supplementary file 18 — Online Resource 18 Dotplots showing genome alignments of cv. Morex (carrying haplotype 1) and cv. Barke (carrying haplotype 2) of a) the entire haplotype region on chromosome 5H illustrating the structural variation at the start and end of the region, b) the start of the haplotype region illustrating an insertion in Barke followed by two inversions further downstream and c) the end of the haplotype region illustrating an insertion in Barke followed by a deletion or potential translocation [file 122_2023_4418_MOESM18_ESM.tiff]

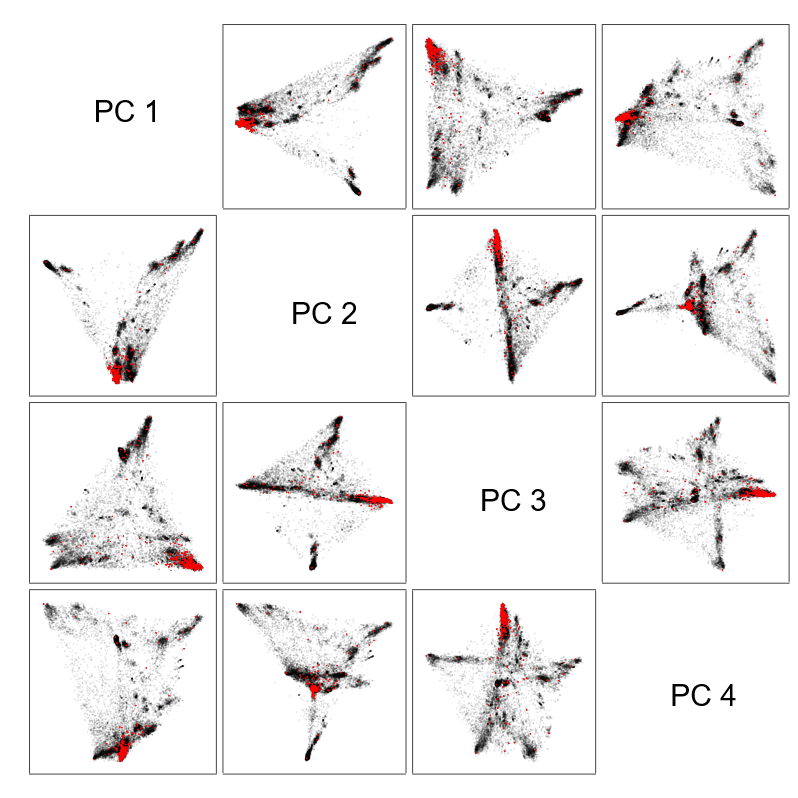

Supplement: Supplementary file 21 — Online Resource 21 PCAs of all domesticated barley accessions of the German Federal ex-situ gene bank at IPK Gatersleben. Red dots represent the 243 accessions carrying the same names as cultivars and landraces from the European two-rowed spring barley panel. The figure was created using the IPK Bridge Portal (IPK Gatersleben - BRIDGE Web Portal (ipk-gatersleben.de) [file 122_2023_4418_MOESM21_ESM.png]

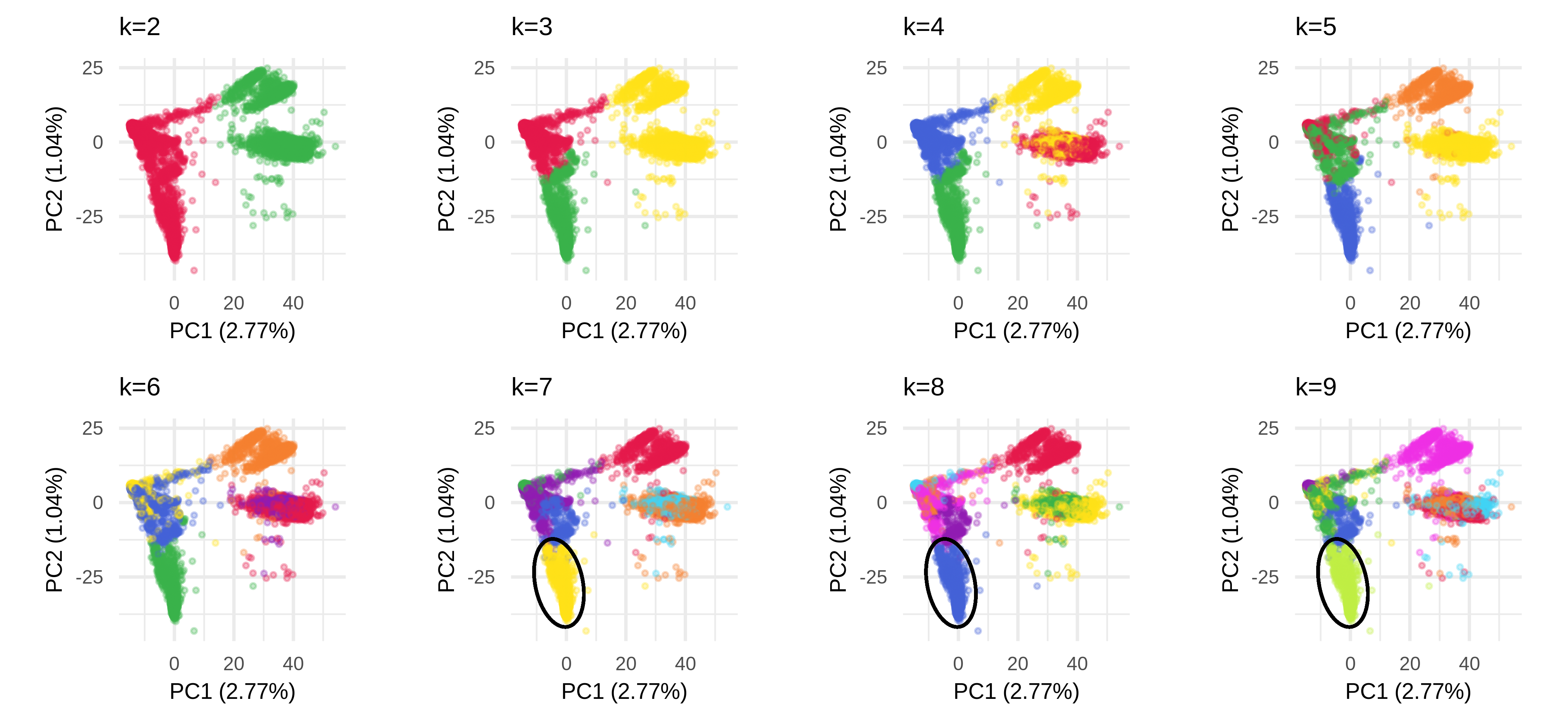

Supplement: Supplementary file 23 — Online Resource 23 Clustering of domesticated barley accessions of the IPK gene bank at different k. While the approach is not suitable to clearly distinguish haplotype 1, haplotype 3 and other potential haplotypes clustering together, haplotype 2 is clearly distinguished at k >= 7 [file 122_2023_4418_MOESM23_ESM.tiff]
