# Supplementary material for: Major chromosome 5H haplotype switch structures the European two-rowed spring barley germplasm of the past 190 years
Source: Theor Appl Genet. 2023 Jul 21;136(8):174. doi: 10.1007/s00122-023-04418-7 (PMC10361897; doi:10.1007/s00122-023-04418-7)
Supplement: Supplementary file 8 — Online Resource 8 Annotation, gene IDs and genetic positions in reference genomes Barke and Morex V3 as well as the reference transcriptome BaRTv2 of the 48 genes differentially expressed in haplotype 1 and 2. These 48 genes form a distinct group in a PCoA of all genes located between 69 and 320 Mbp on chromosome 5H (see Fig. 7, red clusters). All genes were highly expressed in cultivars carrying haplotype 2 except for the two genes marked with asterisks which were highly expressed in cultivars carrying haplotype 1 and low/not expressed in cultivars carrying haplotype 2 [file 122_2023_4418_MOESM8_ESM.docx]

**Major chromosome 5H haplotype switch structures the European two-rowed spring barley germplasm of the past 190 years**

Ronja Wonneberger, Miriam Schreiber, Allison Haaning, Gary J. Muehlbauer, Robbie Waugh, Nils Stein (stein@ipk-gatersleben.de)

Theoretical and Applied Genetics

**Online Resource 8** Annotation, gene IDs and genetic positions in reference genomes Barke and Morex V3 as well as the reference transcriptome BaRTv2 of the 48 genes differentially expressed in haplotype 1 and 2. These 48 genes form a distinct group in a PCoA of all genes located between 69 and 320 Mbp on chromosome 5H (see Fig. 7, red clusters). All genes were highly expressed in cultivars carrying haplotype 2 except for the two genes marked with asterisks which were highly expressed in cultivars carrying haplotype 1 and low/not expressed in cultivars carrying haplotype 2

| BaRTv2 gene | Position | BaRT annotation | Barke gene | Barke annotation | Morex gene | Morex annotation |
| --- | --- | --- | --- | --- | --- | --- |
| BaRT2v18chr5HG228320 | 70332646 -70334017 |  | HORVU.BARKE.r1.5HG0294400 | Nitric oxide reductase FlRd-NAD(+) reductase | HORVU.MOREX.r3.5HG0438640 |  |
| BaRT2v18chr5HG228330 | 70332647 -70333997 |  | HORVU.BARKE.r1.5HG0294400 | Nitric oxide reductase FlRd-NAD(+) reductase | HORVU.MOREX.r3.5HG0438640 |  |
| BaRT2v18chr5HG228710 | 75297443 -75301320 |  | HORVU.BARKE.r1.5HG0294930 | Beta-glucosidase, GBA2 type family protein | HORVU.MOREX.r3.5HG0439370 |  |
| BaRT2v18chr5HG228890 | 77726888 -77730167 |  |  |  |  |  |
| BaRT2v18chr5HG229090 | 81111550 -81113395 |  |  |  |  |  |
| BaRT2v18chr5HG229220 | 84500820 -84502444 |  |  |  |  |  |
| BaRT2v18chr5HG229290 | 85244898 -85246907 |  |  |  |  |  |
| BaRT2v18chr5HG230180 | 101639143 -101647273 |  |  |  |  |  |
| BaRT2v18chr5HG230190 | 101644543 -101651150 | FAR1 domain-containing protein | HORVU.BARKE.r1.5HG0297110 | Protein FAR1-RELATED SEQUENCE 5 |  |  |
| BaRT2v18chr5HG230360 | 103972574 -103976413 |  | HORVU.BARKE.r1.5HG0297340 | ABC transporter B family protein | HORVU.MOREX.r3.5HG0442920 |  |
| BaRT2v18chr5HG231510 | 123922277 -123922931 |  | HORVU.BARKE.r1.5HG0299140 | Neurotoxin alpha-KTx 29.1 | HORVU.MOREX.r3.5HG0445390 |  |
| BaRT2v18chr5HG231520 | 124077553 -124078111 |  | HORVU.BARKE.r1.5HG0299290 | Plant thionin family protein | HORVU.MOREX.r3.5HG0445100 |  |
| BaRT2v18chr5HG231600 | 125421512 -125425321 |  |  |  |  |  |
| BaRT2v18chr5HG231770 | 128751478 -128756130 |  |  |  |  |  |
| BaRT2v18chr5HG231820 | 129341688 -129346395 |  | HORVU.BARKE.r1.5HG0299460 | Biosynthetic peptidoglycan transglycosylase | HORVU.MOREX.r3.5HG0445700 |  |
| BaRT2v18chr5HG231830 | 129640388 -129644430 |  |  |  |  |  |
| BaRT2v18chr5HG232350 | 140622443 -140623548 |  | HORVU.BARKE.r1.5HG0300200 | Late embryogenesis abundant (LEA) hydroxyproline-rich glycoprotein family | HORVU.MOREX.r3.5HG0446860 | Late embryogenesis abundant (LEA) hydroxyproline-rich glycoprotein family |
| BaRT2v18chr5HG232640 | 146087582 -146093946 | IAA-amino acid hydrolase ILR1;IAA-amino acid hydrolase ILR1-like 9 isoform X2 | HORVU.BARKE.r1.5HG0300790 | IAA-amino acid hydrolase ILR1 | HORVU.MOREX.r3.5HG0447690 | IAA-amino acid hydrolase ILR1 |
| BaRT2v18chr5HG233030 | 155397977 -155400120 |  | HORVU.BARKE.r1.5HG0301340 | 6,7-dimethyl-8-ribityllumazine synthase | HORVU.MOREX.r3.5HG0448520 |  |
| BaRT2v18chr5HG233120 | 156549285 -156556626 |  | HORVU.BARKE.r1.5HG0301490 | Serine/threonine-protein kinase RIO1 | HORVU.MOREX.r3.5HG0448700 |  |
| BaRT2v18chr5HG233570* | 171065170 -171065518 |  |  |  |  |  |
| BaRT2v18chr5HG233580* | 171072782 -171073253 |  |  |  |  |  |
| BaRT2v18chr5HG233620 | 172069209 -172073309 |  |  |  | HORVU.MOREX.r3.5HG0450000 |  |
| BaRT2v18chr5HG234000 | 188609100 -188612071 |  |  |  |  |  |
| BaRT2v18chr5HG234400 | 205600458 -205605322 |  | HORVU.BARKE.r1.5HG0336290 | Chromatin assembly factor-1 |  |  |
| BaRT2v18chr5HG235150 | 234382968 -234388762 | Putative zinc finger, CCHC-type |  |  |  |  |
| BaRT2v18chr5HG235160 | 234389412 -234395262 |  |  |  |  |  |
| BaRT2v18chr5HG235610 | 246015651 -246017169 |  |  |  | HORVU.MOREX.r3.5HG0457120 |  |
| BaRT2v18chr5HG235670 | 246756461 -246759659 |  | HORVU.BARKE.r1.5HG0289360 | Cullin-associated NEDD8-dissociated protein 1 |  |  |
| BaRT2v18chr5HG236300 | 262358395 -262361748 |  | HORVU.BARKE.r1.5HG0305930 | C2H2-type zinc finger family protein | HORVU.MOREX.r3.5HG0457260 |  |
| BaRT2v18chr5HG236630 | 273386174 -273397524 |  |  |  |  |  |
| BaRT2v18chr5HG236670 | 273713414 -273716200 |  |  |  |  |  |
| BaRT2v18chr5HG236830 | 277258633 -277262353 |  |  |  |  |  |
| BaRT2v18chr5HG236990 | 281576073 - 281578818 |  | HORVU.BARKE.r1.5HG0307020 | Disease resistance protein (CC-NBS-LRR class) family |  |  |
| BaRT2v18chr5HG237450 | 293586240 -293591515 |  | HORVU.BARKE.r1.5HG0307680 | cytochrome P450, family 709, subfamily B, polypeptide 1 | HORVU.MOREX.r3.5HG0460070 |  |
| BaRT2v18chr5HG237470 | 294195531 -294206537 | Retrotransposon gag domain-containing protein (Fragment) | HORVU.BARKE.r1.5HG0307710 | Transposable element protein, putative, Retrotrans_gag |  |  |
| BaRT2v18chr5HG238070 | 307738123 -307751403 |  |  |  | HORVU.MOREX.r3.5HG0461420 |  |
| BaRT2v18chr5HG238320 | 311836606 -311839556 | iron-sulfur assembly protein IscA-like 2, mitochondrial;Iron-sulfur assembly protein IscA-like 2, mitochondrial;FeS cluster insertion protein | HORVU.BARKE.r1.5HG0308920 | Iron-sulfur cluster insertion protein ErpA | HORVU.MOREX.r3.5HG0473180 | Iron-sulfur cluster insertion protein ErpA |
| BaRT2v18chr5HG238370 | 312353556 -312355285 |  | HORVU.BARKE.r1.5HG0309100 | PP2A regulatory subunit TAP46 | HORVU.MOREX.r3.5HG0473320 | Amino acid dehydrogenase family protein |
| BaRT2v18chr5HG238380 | 312463555 -312468412 |  | HORVU.BARKE.r1.5HG0309120 | DegP protease-like | HORVU.MOREX.r3.5HG0481310 | AT-rich interactive domain-containing protein 4 |
| BaRT2v18chr5HG238390 | 314005474 -314006381 |  | HORVU.BARKE.r1.5HG0309160 | Histone H2A | HORVU.MOREX.r3.5HG0499250 | Histone H2A |
| BaRT2v18chr5HG238400 | 314912218 -314916822 |  | HORVU.BARKE.r1.5HG0309170 | Carboxypeptidase |  |  |
| BaRT2v18chr5HG238410 | 315188210 -315191171 |  | HORVU.BARKE.r1.5HG0309180 | S-adenosyl-L-methionine-dependent methyltransferases superfamily protein |  |  |
| BaRT2v18chr5HG238440 | 315868363 -315872526 | 9-cis-epoxycarotenoid dioxygenase, putative;9-cis-epoxycarotenoid dioxygenase NCED2, chloroplastic-like;Carotenoid oxygenase | HORVU.BARKE.r1.5HG0316630 | Carotenoid cleavage dioxygenase | HORVU.MOREX.r3.5HG0473380 | Carotenoid cleavage dioxygenase |
| BaRT2v18chr5HG238450 | 315882251 -315883824 |  | HORVU.BARKE.r1.5HG0309240 | F-box protein-like protein | HORVU.MOREX.r3.5HG0473390 | F-box family protein |
| BaRT2v18chr5HG238630 | 317452517 -317464191 |  |  |  | HORVU.MOREX.r3.5HG0440940 |  |
| BaRT2v18chr5HG238640 | 317467589 -317471071 |  |  |  |  |  |
| BaRT2v18chr5HG238840 | 319905155 -319908848 | RMUA;SWIM-type domain-containing protein;Transposon protein, putative, Mutator sub-class | HORVU.BARKE.r1.5HG0309740 | Transposon protein, putative, Mutator sub-class | HORVU.MOREX.r3.5HG0462980 |  |

* Gene expression is downregulated in haplotype 2 compared to haplotype 1
