# Supplementary material for: Major chromosome 5H haplotype switch structures the European two-rowed spring barley germplasm of the past 190 years
Source: Theor Appl Genet. 2023 Jul 21;136(8):174. doi: 10.1007/s00122-023-04418-7 (PMC10361897; doi:10.1007/s00122-023-04418-7)
Supplement: Supplementary file 20 — Online Resource 20 GO enrichment of the 1080 genes located between 68.78 and 320.04 Mbp on chromosome 5H. Shown are the GO IDs, GO terms, the number of annotated genes per term in BartV2.0, the number of genes observed per term, the number of genes expected per term, the p-value of gene enrichment and the GO category [file 122_2023_4418_MOESM20_ESM.docx]

**Major chromosome 5H haplotype switch structures the European two-rowed spring barley germplasm of the past 190 years**

Ronja Wonneberger, Miriam Schreiber, Allison Haaning, Gary J. Muehlbauer, Robbie Waugh, Nils Stein (stein@ipk-gatersleben.de)

Theoretical and Applied Genetics

**Online Resource 20** GO enrichment of the 1080 genes located between 68.78 and 320.04 Mbp on chromosome 5H. Shown are the GO IDs, GO terms, the number of annotated genes per term in BartV2.0, the number of genes observed per term, the number of genes expected per term, the p-value of gene enrichment and the GO category

| GO ID | Term | Annotated | Observed | Expected | Weighted Fisher* | Category |
| --- | --- | --- | --- | --- | --- | --- |
| GO:0005516 | Calmodulin binding | 157 | 15 | 3.64 | 3.9e-06 | MF |
| GO:0010427 | Abscisic acid binding | 29 | 6 | 0.67 | 4.5e-05 | MF |
| GO:0004864 | Protein phosphatase inhibitor activity | 40 | 6 | 0.93 | 0.3e-04 | MF |
| GO:0080163 | regulation of protein serine/threonine phosphatase activity | 25 | 6 | 0.58 | 1.9e-05 | BP |
| GO:0032515 | negative regulation of phosphoprotein phosphatase activity | 40 | 6 | 0.93 | 0.00031 | BP |
| GO:0080142 | regulation of salicylic acid biosynthetic process | 18 | 4 | 0.42 | 0.00069 | BP |

*Fisher test, “weighted01” method in the R package TopGo
